# A partial genome assembly of the miniature parasitoid wasp, *Megaphragma amalphitanum*

**DOI:** 10.1371/journal.pone.0226485

**Published:** 2019-12-23

**Authors:** Fedor S. Sharko, Artem V. Nedoluzhko, Brandon M. Lê, Svetlana V. Tsygankova, Eugenia S. Boulygina, Sergey M. Rastorguev, Alexey S. Sokolov, Fernando Rodriguez, Alexander M. Mazur, Alexey A. Polilov, Richard Benton, Michael B. Evgen'ev, Irina R. Arkhipova, Egor B. Prokhortchouk, Konstantin G. Skryabin

**Affiliations:** 1 Institute of Bioengineering, Research Center of Biotechnology of the Russian Academy of Sciences, Moscow, Russia; 2 National Research Center “Kurchatov Institute”, Moscow, Russia; 3 Nord University, Faculty of Biosciences and Aquaculture, Bodø, Norway; 4 Josephine Bay Paul Center for Comparative Molecular Biology and Evolution, Marine Biological Laboratory, Woods Hole, Massachusetts, United States of America; 5 Lomonosov Moscow State University, Faculty of Biology, Moscow, Russia; 6 Center for Integrative Genomics, Faculty of Biology and Medicine, University of Lausanne, Lausanne, Switzerland; 7 Institute of Molecular Biology RAS, Moscow, Russia; Hospital for Sick Children, CANADA

## Abstract

Body size reduction, also known as miniaturization, is an important evolutionary process that affects a number of physiological and phenotypic traits and helps animals conquer new ecological niches. However, this process is poorly understood at the molecular level. Here, we report genomic and transcriptomic features of arguably the smallest known insect–the parasitoid wasp, *Megaphragma amalphitanum* (Hymenoptera: Trichogrammatidae). In contrast to expectations, we find that the genome and transcriptome sizes of this parasitoid wasp are comparable to other members of the Chalcidoidea superfamily. Moreover, compared to other chalcid wasps the gene content of *M*. *amalphitanum* is remarkably conserved. Intriguingly, we observed significant changes in *M*. *amalphitanum* transposable element dynamics over time, in which an initial burst was followed by suppression of activity, possibly due to a recent reinforcement of the genome defense machinery. Overall, while the *M*. *amalphitanum* genomic data reveal certain features that may be linked to the unusual biological properties of this organism, miniaturization is not associated with a large decrease in genome complexity.

## Introduction

**Fig 1:**
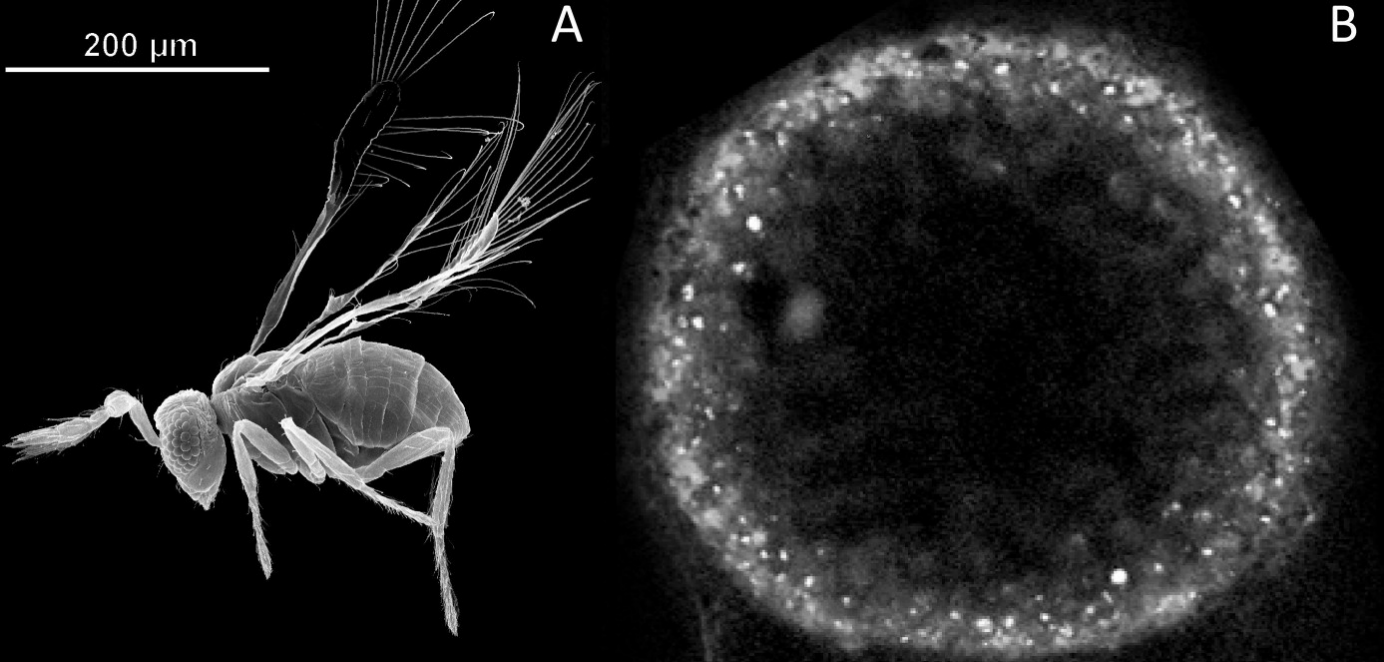
Size comparison of the parasitoid wasp *M*. *amalphitanum* and bacterium *Thiomargarita namibiensis*. (A) An adult stage of the parasitoid wasp *M*. *amalphitanum* (image adapted from [5]), (B) *T*. *namibiensis*–the largest known bacterium (modified from Schulz et al. 1999) [11].

Miniaturization in animals is an evolutionary process that is frequently accompanied by structural simplification and size reduction of organs, tissues and cells [1, 2]. The parasitoid wasp *Megaphragma amalphitanum* (Hymenoptera: Trichogrammatidae, subfamily Oligositinae) is one of the smallest known insects, whose size (250 μm adult length) is comparable with unicellular eukaryotes and even some bacteria (Fig 1). Parasitoids from the genus *Megaphragma* parasitize greenhouse thrips *Heliothrips haemorrhoidalis* (Thysanoptera: Thripidae) developing on the shrubs *Viburnum tinus* (Adoxaceae) and *Myrtus communis* (Myrtaceae) [3], and possibly *Hercinothrips femoralis* (Thysanoptera: Thripidae) [4]. The wasp spends most of its life cycle in host eggs, while the imago stage is very short and lasts only a few days [3, 4]. *M*. *amalphitanum* belongs to chalcid wasps, which represent one of the largest insect superfamilies (~23,000 described species)[5]. The higher-level taxonomic relationships of Trichogrammatidae, Chalcidoidea and Hymenoptera have been investigated in several recent studies [6–10] that helped to establish the placement of this unique taxon that related to Mymaridae and Pteromalidae.

Amongst notable anatomical features of *M*. *amalphitanum*, this species has only ~4,600 neurons in its brain, which is substantially fewer than in the brains of other wasps, e.g., the parasitoid chalcid wasp *Trichogramma pretiosum* (Trichogrammatidae: Trichogrammatinae) (~18,000 neurons), *Hemiptarsenus sp*. (Chalcidoidea: Eulophidae) (~35,000 neurons), and the honey bee *Apis mellifera* (Apidae) (~850,000–1,200,000 neurons). Moreover, by the final stage of *M*. *amalphitanum* development, up to 95 percent of the neurons of the central nervous system have lost their nuclei [12, 13]. Nevertheless, adult wasps, which have an average lifespan of 5 days, still preserve the basic functional traits of hymenopteran insects including flight, mating and oviposition in hosts [14].

In this study, we present a *M*. *amalphitanum* partial genome assembly and the adult transcriptome, and compare these with several parasitoid wasp species of different body sizes from the Chalcidoidea and Ichneumonoidea hymenopteran superfamilies. We performed general gene ontology and pathway analyses as well as specific gene categories of interest, such as chemosensory receptors and venom components. Additionally, we investigated transposable element (TE) content and dynamics across *M*. *amalphitanum* and other parasitoid wasp species and analyzed the major components of the genome defense machinery. As body size reduction and loss of physiological or phenotypic traits is often correlated with genome size diminution [15, 16] and/or gene networks reduction [17], including chromatin diminution from the somatic tissues during embryogenesis[18, 19], we initially anticipated that the *M*. *amalphitanum* genome would be greatly simplified.

## Material and methods

### Detailed information is presented in *Supplementary Information*

#### Nucleic acid extraction and library construction

*M*. *amalphitanum* individuals were reared in the laboratory conditions from eggs of *Heliothrips haemorrhoidalis* (Thysanoptera: Thripidae) collected in Santa Margherita, Northern Italy (44.32, 9.20). Unfortunately, we could collect only a dozen *M*. *amalphitanum* individuals because their habitats are difficult to detect (culture in the laboratory is currently impossible), the imago life span is short (5 days), and the animal is extremely small. With several insects we could cleanly recover, we were therefore able to obtain only around 1–5 ng of genomic DNA for the each paired-end DNA and cDNA libraries. DNA was extracted from ten individuals (males and females) using NucleoSpin Tissue XS kit (Macherey-Nagel, Germany) for each DNA-library. Three DNA libraries (DNA-library1 –whole insects; DNA-library2 –thorax and abdomen; DNA-library3 –head) were constructed using Ovation Ultralow Systems V2 kit (NuGEN, USA). Limited amount of biological material and low quantity of starting material (1–3 ng) did not permit construction of mate-paired libraries. Genome libraries were sequenced using Illumina HiSeq 1500 (Illumina, USA) with 150 bp paired-end reads. RNA was extracted from ten *M*. *amalphitanum* individuals (males and females) using the Trizol reagent (Thermo Fisher Scientific, USA) by a standard protocol, and cDNA libraries were constructed using Ovation RNA-Seq System V2 kit (NuGEN, USA) with poly(A) enrichment.

#### Genome de novo assembly

The output from Illumina sequencing of the genomic DNA library (source format *.fastq) was used for *de novo* genome assembly. To assemble the genome of *M*. *amalphitanum*, we used 102,188,833 paired-end reads. Genome assemblies were constructed using different assembly algorithms, and their performance was compared to each other (S2 Fig). Then, *M*. *amalphitanum* reads were mapped to the final partial assembly with 92.3% conformity. Additionally, genomic DNA-libraries from thorax and abdomen (DNA-library2) of *M*. *amalphitanum* (SRR5982987) and from head (DNA-library3) of *M*. *amalphitanum* (SRR5982986) were prepared. In total, 79,317,970 (paired-end sequencing: 2×100 bp) and 85,409,775 (single-end sequencing: 50 bp) DNA reads were sequenced and used for *M*. *amalphitanum* coverage increase and as additional evidence during the search for potentially missing genes (S1 Table). Then, these reads were used for *de novo* building of the *M*. *amalphitanum* genome sequence by the SPAdes assembler (v.3.6.1) [20].

#### Transcriptome de novo assembly

Illumina RNA sequencing generated a total of 59,790,973 paired-end reads. Transcriptome *de novo* assembly was conducted using the default k-mer size in the Trinity software package (v. 2.4.0) [21], which combines three assembly algorithms: Inchworm, Chrysalis and Butterfly. Annotation of the *M*. *amalphitanum* transcriptome assembly was performed using the Trinotate pipeline [22].

*Transposable element (TE) de novo identification and analysis*. For *de novo* TE library construction, we used the REPET package [23] which combines three mutually complementing repeat identification tools (RECON, GROUPER and PILER), yielding a combined repeat library with the average consensus sequence length of 1.66 kb (ranging from 157–14,640 bp). The outputs were subject to additional classification with the RepeatClassifier tool from the RepeatMasker package (www.repeatmasker.org), which was also used to build the corresponding TE landscape divergence plots.

## Results and discussion

### Genome and transcriptome sequencing and assembly of *M*. *amalphitanum*

To gain insight into the genomic signatures of miniaturization that would distinguish *M*. *amalphitanum* from other Hymenoptera, we performed whole-genome shotgun sequencing of DNA (DNA-library1) isolated from ten adult individuals (males and females), using the Illumina platform **(**S1 Table**).** The resulting partial genome assembly (PRJNA344956) has a cumulative length of 346 megabases (Mb), with a scaffold N50 of 10,296 bp. The total genome coverage is 88.6-fold. Thus, the genome of *M*. *amalphitanum* is comparable in size with other Chalcidoidea wasps, such as *Copidosoma floridanum*, *T*. *pretiosum* or *Nasonia vitripennis* [24, 25]. The best-performing combination of assembly software yielded contig N50 of 4,285 bp and allowed us to assemble 94,687 scaffolds from the low amounts of starting DNA material (Table 1; S2 Table; S1 Fig).

**Table 1:** Final statistics of the genome and transcriptome assemblies of parasitoid wasp *Megaphragma amalphitanum*.

**Genome assembly**	
**Number of contigs**	94,687
**Median (n:N50)**	7,843
**Contig N50 size**	10,296
**Maximum contig length, bp**	895,906
**Cumulative assembly size, bp**	3.46×10^8^
**BUSCO assembly completeness, %** **Fragmented, %**	80.4 9.8
**Transcriptome assembly**	
Number of contigs	46,841
Median (n:N50)	13,109
Contig N50 size	633
Maximum contig length, bp	9,503
Cumulative assembly size, bp	3.74×10^7^
BUSCO assembly completeness, % Fragmented, %	24.65 28.12
Number of transcripts **(**BLASTX**)**	12,238

The *M*. *amalphitanum* genome assemblies were evaluated with the BUSCO v3 (benchmarking universal single-copy orthologs) Hymenoptera gene set [26], which uses 4,415 near-universal single-copy orthologs to assess the relative completeness of genome assemblies. Through this analysis, 7.55% of the conserved genes were initially identified in the *M*. *amalphitanum* assembly as putatively missing (S3 Table). More detailed information on our extensive search for the missing genes in *M*. *amalphitanum* genome is presented below.

We also performed whole-body transcriptome analysis using RNA extracted from ten *M*. *amalphitanum* individuals (males and females). Transcriptome *de novo* assembly (PRJNA344956) was performed using the Trinity software [21]. A total of 46,841 contigs were assembled with a mean length of 586 bp and an N50 of 633 bp from the low amounts of starting RNA material (S4 Table**)**. The Illumina paired-end RNA-Seq data from *M*. *amalphitanum* were mapped to the previously assembled genome using Bowtie2 [27]. Inspection of the alignments revealed that 79.95% of reads could be mapped to the genome. The BUSCO v3 statistics for the transcriptome assembly is also presented in Table 1; S3 Table.

The BUSCO analysis shows the low completeness of the present partial genome and transcriptome assemblies, with 28–29% of BUSCO genes listed as fragmented. This may be caused by inability to use mate-paired DNA-libraries or single-molecule sequencing (because of low amount of starting DNA material); possible high heterozygosity and/or significant structural variation between different parasitoid wasp individuals that were used for genome and transcriptome assemblies; BUSCO database incompleteness; and other factors. An additional factor in poor transcriptome completeness could be a high number of short and chimeric isotigs: while nearly 80% of transcriptome reads map to the genome, only 24% of assembled contigs are represented in the complete BUSCO set.

### Gene ontology analysis

We used Gene Ontology (GO) analysis terms to describe characteristics of *M*. *amalphitanum* gene products in three independent categories: biological processes (S2 Fig), molecular function (S3 Fig), and cellular components (S4 Fig). BLASTX outputs were used to retrieve the associated gene names and GO terms in all three categories (Table 2).

**Table 2:** Basic Gene Ontology (GO) analysis terms for *M*. *amalphitanum* gene products.

GO assignments of the transcripts	Transcript counts and percentage of total		
Biological processes	8,812 counts, 49.72%		
	Transcription	Regulation of transcription	DNA integration
	15%	10%	8%
Cellular components	4,802 counts, 27.10%		
	Nucleus and cytoplasm components	Integral membrane components	Plasma membrane components
	18%	9%	7%
Molecular functions	4,108 counts, 23.18%		
	ATP binding	Metal ion binding	Zinc ion binding
	17%	12%	10%

All *M*. *amalphitanum* transcripts were matched to the Clusters of Orthologous Groups (COG) database to predict and classify their functions. In total, 8,810 genes were assigned to 25 COG functional categories. One of the largest groups is represented by the cluster for post-translational modification, protein turnover, and chaperones (988 counts; 10.7%), followed by intracellular trafficking, secretion, and vesicular transport (659 counts; 7.2%), DNA replication, recombination and repair (606 counts; 6.6%), signal transduction mechanisms (599 counts, 6.5%) and transcription (587; 6.4%) (S5 Fig).

To better understand incorporation of genes into diverse pathways, all annotated transcripts were mapped against the KEGG database for pathway-based analysis. As a result, 6,130 transcripts out of a total of 46,841 were assigned to a KEGG pathway, and were present in 328 different KEGG pathways. The KEGG pathway distribution is summarized in S6 Fig. The top pathways are biosynthesis of secondary metabolites (150 counts; 2.4%), RNA transport (100 counts; 1.6%), biosynthesis of antibiotics (95 counts; 1.5%), and spliceosome (94 counts; 1.5%).

The annotation of *M*. *amalphitanum* and the available transcriptome assemblies of other parasitoid wasps from the families Trichogrammatidae (*T*. *pretiosum*, a lepidopteran egg parasitoid) and Braconidae including *Cotesia vestalis* (a diamondback moth parasitoid), *Diachasma alloeum* (an apple maggot parasitoid) and *Fopius arisanus* (tephritid fruit fly parasitoid) were used for comparative analysis of the most represented gene functions in parasitoids. We also used transcriptome assemblies from the Agaonidae fig wasp, *Ceratosolen solmsi*. We found significant similarities between *M*. *amalphitanum*, *T*. *pretiosum* and *C*. *vestalis* major GO enrichment categories (S7–S9 Figs). At the same time, a significant number of transcripts related to DNA integration relative to other parasitoid wasps was found in *D*. *alloeum* and *M*. *amalphitanum* (S7 Fig) (see below). Complete information about reference datasets used for *M*. *amalphitanum* genome and transcriptome data analysis is shown in S5 Table. The Trinotate statistics for annotation of *M*. *amalphitanum*, *C*. *solmsi*, *D*. *alloeum*, *F*. *arisanus*, *C*. *vestalis* and *T*. *pretiosum* transcriptome assemblies is presented in S6 Table.

### Potentially missing genes and missing or rapidly evolving gene clusters in the *M*. *amalphitanum* genome

Given the incomplete nature of the *M*. *amalphitanum* genome assembly (BUSCO coverage of ~80%, Table 1), we could perform only a preliminary assessment of potentially missing genes and/or rapidly evolving gene clusters compared to other species. We clustered gene orthologs and identified gene clusters for each hymenopteran taxa (Chalcidoidea: *M*. *amalphitanum*, *T*. *pretiosum*, *C*. *solmsi*, C. *floridanum*, and *N*. *vitripennis*; Ichneumonoidea: *D*. *alloeum* and *F*. *arisanus*; Apoidea: *A*. *mellifera*) using OrthoMCL [28]. The core gene set of all the hymenopteran species was composed of 6,278 gene clusters, 122 gene clusters were unique to the chalcid clade. 262 gene clusters were not detected in any of the chalcids analyzed (Supplementary Dataset 2; NCBI BioProject: PRJNA344956), but found in all the other hymenopterans, consistent with a similar recent analysis [29]. Our findings suggest that that the loss of these genes apparently occurred in the last common ancestor of chalcids, or point to the possibility of parallel genome evolution across these species. Interestingly, the missing/rapidly evolving genes include homologs of genes that have important roles in embryonic patterning and development in other insects (e.g., *krueppel-1*, *knirps* or *short gastrulation* [29]).

To determine whether miniaturization in *M*. *amalphitanum* is associated with significant gene loss that could be detected even in a partial genome assembly, we used genomic data of six larger hymenopteran species (*T*. *pretiosum*, *C*. *vestalis*, *C*. *floridanum*, *F*. *arisanus*, *N*. *vitripennis*, and *N*. *giraulti*), as well as the well-annotated genome of the honeybee (*A*. *mellifera*) as reference (body sizes are presented in S5 Table**)**. We mapped the *M*. *amalphitanum* (DNA-library1), *T*. *pretiosum*, *C*. *vestalis*, *C*. *floridanum*, *F*. *arisanus*, *N*. *vitripennis*, *N*. *giraulti* DNA reads on the *A*. *mellifera* genome sequence (PRJNA13343, PRJNA10625) (S10 Fig), and detected 115 genes that were not represented by *M*. *amalphitanum* sequencing reads but were present in other parasitoid wasps. We then increased the coverage of the *M*. *amalphitanum* genome to 146.8-fold by adding the reads from additional libraries (DNA-library2 and DNA-library3) (S1 Table) and observed the apparent absence of 114 of the 115 genes. An additional TBLASTX search identified 36 of these genes as present, yielding a total of 78 putatively missing genes (S7 Table). However, querying the *M*. *amalphitanum* genome with the corresponding amino acid sequences from the closest wasp ortholog (*N*. *vitripennis* or *T*. *pretiosum*) in TBLASTN searches reduced the number of putatively missing genes to just five: centrosomin, phosphoglycerate mutase 5, phosphoglycerate mutase 5–2, 26S proteasome complex subunit DSS1, and mucin-1/nucleoporin NSP1-like. We detected short *M*. *amalphitanum* genome sequences encoding protein fragments (~8–23 amino acid residues) with some similarity to four of them, suggesting that they may be in the process of degeneration in this species. Despite a thorough search, we were unable to find any homologous sequence related to *centrosomin (cnn)* gene either in the partially assembled genome or in our cDNA libraries. Although *cnn* is regarded as rapidly evolving [30], sequence homology can be readily discerned and orthologs are present in every other insect, including the parasitoid *T*. *pretiosum*, suggesting that this gene is specifically absent in *M*. *amalphitanum*. In *Drosophila melanogaster*, Cnn has important roles at the centrosome in mitotic spindle formation, cytoskeleton organization and neuronal morphogenesis [31, 32], although these functions may not be indispensable because this species (and possibly other insects) possesses centrosome-independent mechanisms for spindle nucleation [33]. A fungal homolog of Cnn is involved in nuclear migration [34–36]. Since the presented genome assembly has only partial BUSCO coverage, the absence of *cnn* remains tentative. Globally, however, the analysis of the available genome assembly argues for relatively little gene loss in *M*. *amalphitanum*. Confident identification of true gene losses in this species will require additional DNA sequencing and improved genome assembly.

### Chemosensory genes in the *M*. *amalphitanum* genome

Chemosensory receptors are encoded by some of the largest gene families in insect genomes, reflecting their important and wide-ranging roles in detection of environmental odors and tastants. We asked how these gene families have evolved in *M*. *amalphitanum*, whose central and peripheral nervous systems are highly reduced [2, 14]. The highly divergent sequences of chemosensory receptors and relatively short genomic contig lengths available for *M*. *amalphitanum* precluded accurate annotation of full-length sequences in this species for the majority of loci. Nevertheless, comparison with chemosensory receptor repertoires of other insects allowed us to define probable orthologous relationships with receptors of known function in other species and obtain initial estimates of the size of each family.

The most deeply conserved family of chemosensory receptors in insects are the Ionotropic Receptors (IRs), which are distantly related to ionotropic glutamate receptors [37, 38]. IRs function in heteromeric protein complexes comprising more broadly-expressed co-receptors with selectively expressed “tuning” IRs that determines sensory specificity. We identified orthologs of each of the co-receptors (*Ir8a*, *Ir25a* (two paralogs), *Ir93a* and *Ir76b*), as well as four genes encoding tuning IRs related to acid-sensing receptors in other species. We also identified orthologs of IR68a, which functions in hygrosensation [39] and IR21a, which functions in cool temperature-sensing [40, 41]. Overall, the repertoire of IRs in *M*. *amalphitanum* is therefore very similar in size and content to that of *N*. *vitripennis* [38].

Insects possess a second superfamily of chemosensory ion channels–distinguished by a heptahelical protein structure–comprising Odorant Receptor (OR) and Gustatory Receptor (GR) subfamilies, which generally function in detection of volatile and non-volatile stimuli, respectively [42–45]. Similar to IRs, ORs function in heteromeric complexes of a conserved co-receptor (ORCO) and a tuning OR. We identified an *M*. *amalphitanum* ortholog of *Orco* and 83 additional *Or*-related sequences. We caution that many of these *Or* sequences are small fragments (often located near the end of the assembled contigs), so it is currently difficult to determine whether these are intact genes or pseudogenes. Within the GR repertoire, we identified genes encoding proteins related to GR43a, a sensor of both external and internal fructose [46], two others similar to other insect sugar-sensing GRs [47], and 25 additional *Gr* gene fragments. The sizes of these repertoires are smaller than in *N*. *vitripennis* (300 *Or*s (including 76 pseudogenes) and 58 *Gr*s (including 11 pseudogenes) [48]), but similar to non-miniaturized parasitoid wasps *Meteorus pulchricornis* and *Macrocentrus cingulum* [49, 50]. However, precise comparison with the latter two species is difficult, as receptors in these wasps were identified from antennal transcriptomes, thereby representing only one of these insects’ chemosensory organs.

In sum, these analyses reveal that despite drastic nervous system reduction, *M*. *amalphitanum* has retained the conserved chemosensory receptors of larger wasps (and other insects), and appears to have numerous additional order- or species-specific receptors to allow detection of environmental chemical cues.

### Venom components in the *M*. *amalphitanum* transcriptomic data

**Fig 2:**
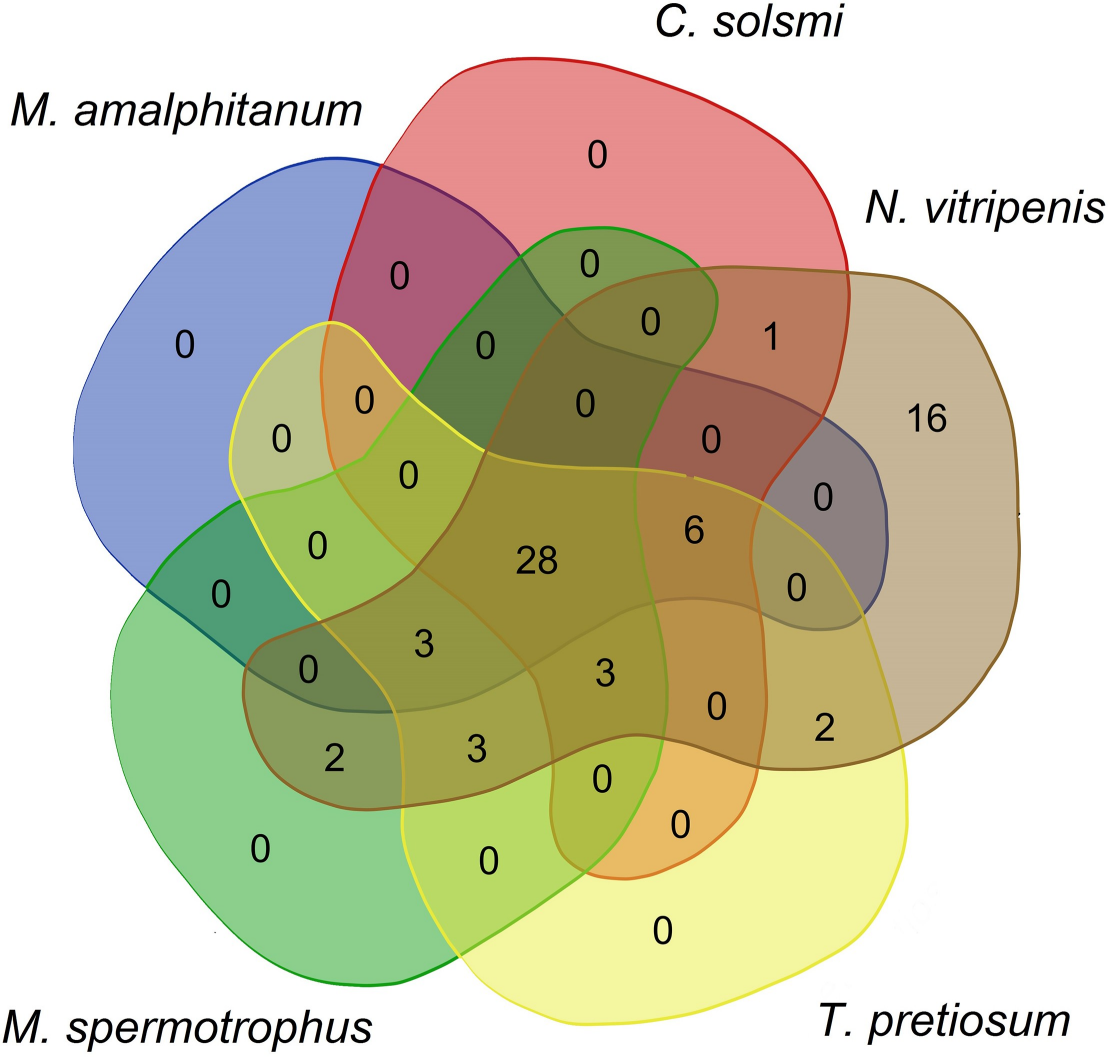
A Venn diagram showing *Nasonia vitripennis* venom components in other Chalcidoidea species: *M*. *spermotrophus*, *C*. *solmsi*, *T*. *pretiosum* and *M*. *amalphitanum*.

Parasitoid wasps often use venom to modify the metabolism of their hosts; toxins and their known or presumed biological functions are described in various species [51]. We investigated the presence of homologs of *N*. *vitripennis* toxin constituents in *M*. *amalphitanum* and other parasitoid wasps (*Megastigmus spermotrophus*, *N*. *vitripennis*, *C*. *solmsi*, *T*. *pretiosum*), using previously published venom data [52, 53] and the transcriptomes of chalcid wasps (S5 Table**).** We identified 28 transcripts encoding putative venom proteins (Fig 2; S8 Table); homologs of these are found in all investigated Chalcidoidea species (Table 3). Assuming that most of these candidates are truly conserved venom proteins among Chalcidoids, *M*. *amalphitanum* venom diversity does not seem to have been significantly affected by size reduction.

**Table 3:** Number of homologs of *N*. *vitripennis* venom (*N*. *vitripennis* toxin constituents) in *M*. *amalphitanum* and other Chalcidoidea species based on Universal Chalcidoidea Database [54].

Parasitoid wasp species	Families of Chalcidoidea	Number of *N*. *vitripennis* venom constituents	Body size, mm	Approximate number of hosts
*M*. *amalphitanum*	Trichogrammatidae	37	0.25	2 insect species from one order
*C*. *solmsi*	Agaonidae	38	2.7	2 plant species from one family
*M*. *spermotrophus*	Torymidae	41	2.8	13 plant species from one family
*T*. *pretiosum*	Trichogrammatidae	45	0.5	>140 insect species from 4 orders
*N*. *vitripennis*	Pteromalidae	64	2.2	6* insect species from one order [55]

* Universal Chalcidoidea Database lists >110 insect species from 8 orders [54]

### *M*. *amalphitanum* transposable elements and genome defense

Transposable elements (TEs) constitute a measurable fraction of virtually all eukaryotic genomes, and can play important roles in their function and evolution. In insects, TE activity has been implicated in evolution of eusociality, based on comparison of ten bee genomes with increasing degrees of social complexity [56]. We performed *de novo* TE identification and comparative analysis of TE dynamics in *M*. *amalphitanum* and in a representative set of larger wasp genomes for which TE content has previously been reported: the parasitoid *N*. *vitripennis* and two primitively eusocial aculeate wasps *Polistes canadensis* and *Polistes dominula* [12, 25, 57]. Additionally, we analyzed TEs in the genomes of parasitoid wasps *T*. *pretiosum* from the family Trichogrammatidae and *D*. *alloeum* from the family Braconidae.

**Fig 3:**
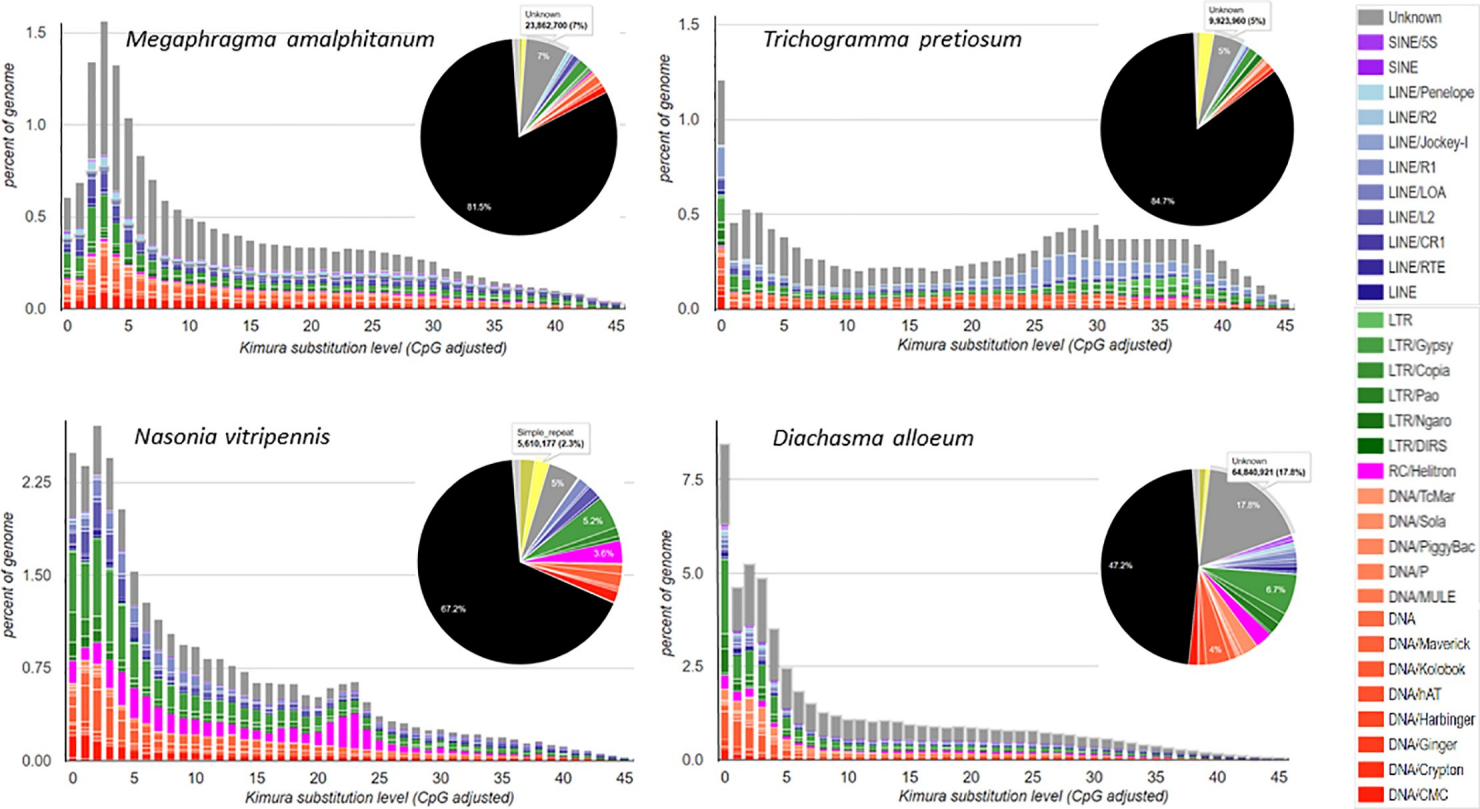
Comparison of TE landscape divergence plots and TE genome fraction pie charts in four parasitoid wasp species: *M*. *amalphitanum*, *T*. *pretiosum*, *N*. *vitripennis* and *D*. *alloeum*.

For uniformity of measurements, we applied the same workflow to all genomes, without relying on pre-existing repeat libraries. We employed the REPET package for *de novo* TE identification (also used in [56]), and RepeatMasker for repeat classification and construction of TE landscape divergence plots. Comparison of the overall repeat content across six wasp species did not reveal substantial differences between four species (18.5% in *M*. *amalphitanum vs*. 18.1%, 17.7% and 14.2% in *P*. *canadensis*, *P*. *dominula* and *T*. *pretiosum*, respectively). The *N*. *vitripennis* genome was 32.5% repetitive, in close agreement with the published estimate [25], and *D*. *alloeum* was highly repetitive at 52.8% (pie charts in Fig 3; S11 Fig). TE dynamics over time, which is shown on the corresponding TE landscape divergence plots, was found to differ substantially for *M*. *amalphitanum*, which displayed a pronounced decline in recent TE activity after an initial increase, a pattern that is rarely observed in other hymenopterans [58, 59] (Fig 3).

While TE dynamics may be affected by different factors, the observed drop in active TE content in *M*. *amalphitanum* may be relevant to the unique biology of this highly miniaturized insect. Its closest relative, *T*. *pretiosum*, is about 2-fold larger in body length. *Wolbachia* infection, which typically results in *T*. *pretiosum* parthenogenesis, can afterwards indirectly affect TE mobility in the host as a consequence of asexual reproduction, resulting in proliferation of specific TE families [58, 60, 61]. Other wasps do not display notable drops or spikes in current TE activity; TE inactivation was reported in two asexual mites [58], however it appears to be ancient and may have occurred prior to the abandonment of sex. Overall, the continued decline in *M*. *amalphitanum* TE activity over the span of several million years–not observed in *T*. *pretiosum* which shares the most recent common ancestor with *M*. *amalphitanum*–represents a rather unusual genomic feature compared to other hymenopteran we examined, including ants (not shown). We note, however, a recent comprehensive study [59] described two hymenopterans with a similar decline in recent TE activity (see below). No traces of *Wolbachia* infection or other representatives of the Rickettsiaceae family were found in *M*. *amalphitanum* individuals [62], while the sequenced *T*. *pretiosum* carries the *Wolbachia* symbiont [63]; the sequenced *Nasonia* strain was maintained on antibiotics to cure it of infection.

**Fig 4:**
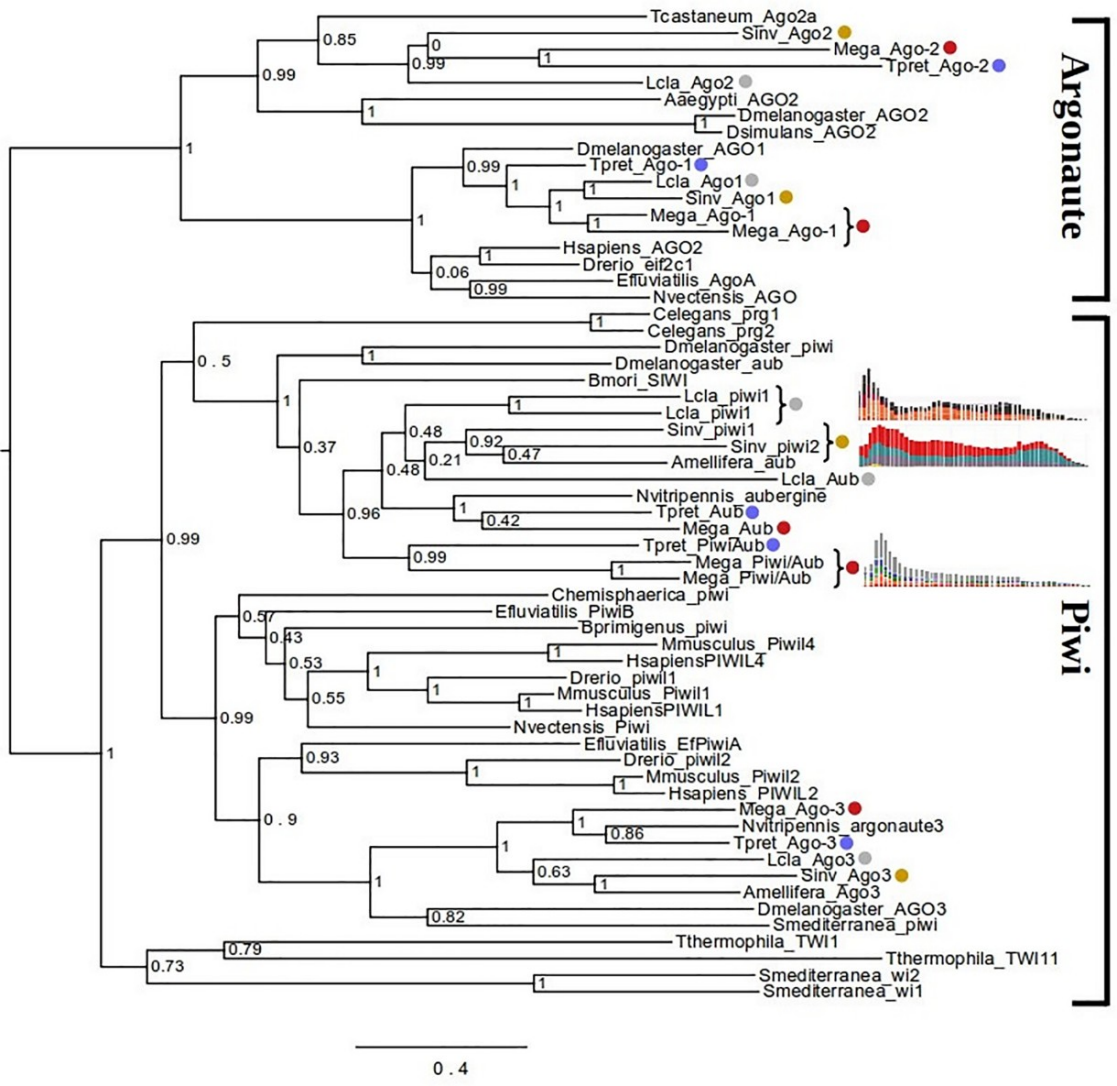
Maximum likelihood analysis of phylogenetic relationships between Piwi/Argonaute coding sequences. Colored dots denote sequences from *T*. *pretiosum* (blue), *L*. *clavipes* (gray), *S*. *invicta* (yellow) and *M*. *amalphitanum* (red). Recent duplications in the latter three hymenopterans are indicated by curly brackets, and the corresponding TE divergence plots from [58, 59] and Fig 3 are placed next to each curly bracket. Phylogeny analysis and notations are as in S12 Fig.

To gain insights into possible reasons for reduction in TE activity after the initial burst, we investigated the major components of the genome defense machinery in *M*. *amalphitanum*, including Dicer (Dcr)-like and Argonaute (Ago)/Piwi-like protein-coding genes. In insects, *Ago-1* and *Dcr-1* homologs represent the key components of the miRNA pathway; *Ago-2* and *Dcr-2* mediate antiviral RNA interference; and *Piwi* and *Ago-3*/*Aub* suppress TE activity in the germline [64]. Both *M*. *amalphitanum* and *T*. *pretiosum* possess equal numbers of *Dcr-1* and *Dcr-2* homologs, as well as *Ago-2* and *Ago-3* homologs (S12 Fig). However, in *M*. *amalphitanum*, the *Ago-1* and the *Piwi/Aub* homologs underwent a relatively recent duplication in comparison to *T*. *pretiosum* (Fig 4). This may indicate additional layers of enforcement in the miRNA and piRNA pathways of *M*. *amalphitanum*, both of which should result in suppression of TE activity. Indeed, after inspecting the genomes of two other sequenced hymenopteran species showing recent declines in TE activity (*Leptopilina clavipes* and *Solenopsis invicta*; [58, 59]), we found that they also display relatively recent duplications of Piwi-like proteins (Fig 4).

The drop in TE activity is also evident from the transcriptome analysis. The GO radar plot (S7 Fig) shows a substantial number of short contigs related to DNA integration, most of which upon inspection were found to represent separate fragments of gypsy-like and copia-like LTR retrotransposons, and a few belong to Polinton, P and Ginger DNA TEs. Transcriptionally active copies fall into two groups: first, those which apparently proliferated during the burst of TE activity and have since accumulated debilitating mutations making them incapable of transposition, but still retain a certain level of transcriptional activity; second, those that originate from recent infections by retrovirus-like TEs and contain uninterrupted ORFs, but are not actively proliferating and are present at very few genomic loci. Comparison of BLASTN hits for *M*. *amalphitanum* integrase-related TE transcripts showed that high-copy hits represent MITEs (S13 Fig). We hypothesize that actively proliferating TE copies represent recent arrivals, possibly brought about by viruses or host-parasite interactions [65].

## Concluding remarks

Our study provides a first view of the genomic content of one of the smallest insects currently known, the parasitoid wasp *M*. *amalphitanum*. In contrast to the expectation that the small body size, in combination with the parasitic lifestyle, should lead to significant reduction in the amount of genomic DNA and in gene content, we do not observe a drastic reduction in the overall genome size or in the number of expressed genes in comparison with larger parasitic wasps. However, the multiple experimental constraints described above limit the quality of genome and transcriptome assemblies. In the future, improved genomic studies in this species (and other Hymenoptera) will be essential to confidently assess specific genetic adaptations that may be linked with body miniaturization.

Interestingly, transposable element dynamics over time were found to differ substantially between the analyzed wasp species, with *M*. *amalphitanum* displaying a relatively recent decline in TE activity preceded by a burst, a pattern not observed in most other parasitoid wasps. The decline in TE activity may have been associated with evolution of additional *Ago* and *Piwi* copies, not present in *T*. *pretiosum*, which could have reinforced the genome defense machinery to prevent uncontrolled TE expansion. This hypothesis is strengthened by identifying duplications of Piwi-like proteins accompanied by a decline in TE activity over time in two additional species of Hymenoptera; by contrast, most other hymenopterans show no such decline.

The relationship between body size and genome size has been discussed for a long time. Significant correlations of these values have been described for flatworms and copepods [16]; by contrast, such correlations were not found in ants [66]. Our results show that body size reduction in hymenopterans is not accompanied by greatly decreased transcriptomic and genomic complexity. This observation begs the question of how miniaturization is encoded genetically. We hypothesize that changes in regulatory sequences, rather than gene content, were important in the process of body size reduction, similar to mechanisms of morphological evolution that have driven adaptive diversification in all animals, great or small [67].

## Supporting information

S1 Fig *M. amalphitanum* genome assembly statistics using ABySS, SPAdes, and Velvet software. K-mer sizes were matched for ABySS, SPAdes and Velvet. Note: CLC Genomics Workbench does not use k-mer size; CLC assembly was performed with default settings, and the statistics are given in S2 Table.
(PNG)

S2 Fig Gene ontology analysis of *M. amalphitanum* transcriptome for contigs with assigned GO: Biological processes. (TIF)

S3 Fig Gene ontology analysis of *M. amalphitanum* transcriptome for contigs with assigned GO: Molecular function. (TIF)

S4 Fig Gene ontology analysis of *M. amalphitanum* transcriptome for contigs with assigned GO: Cellular components. (TIF)

S5 Fig The Clusters of Orthologous Groups (COG) for M. amalphitanum transcriptome (top pathways). (TIF)

S6 Fig KEGG pathway analysis for the *M. amalphitanum* transcriptome. (TIFF)

S7 Fig Radar plot for the *M. amalphitanum, C. solmsi, D. alloeum, F. arisanus, C. vestalis, T. pretiosum* transcriptome GO-category related to biological processes showing numbers of transcripts in this GO-category for six Chalcidoid species. (TIF)

S8 Fig Radar plot for the *M. amalphitanum, C. solmsi, D. alloeum, F. arisanus, C. vestalis, T. pretiosum* transcriptome GO-category related to cellular components showing numbers of transcripts in this GO-category for six Chalcidoid species. (TIF)

S9 Fig Radar plot for the *M. amalphitanum, C. solmsi, D. alloeum, F. arisanus, C. vestalis, T. pretiosum* transcriptome GO-category related to molecular processes showing numbers of transcripts in this GO-category for six Chalcidoid species. (TIF)

S10 Fig Potentially missing genes in the *M. amalphitanum* partial genome assembly. Y-axis: number of genes; X-axis: number of hymenopteran genomes analysed.
(TIF)

S11 Fig Effects of re-classification of “unknown” repeats in the *de novo* library for *M. amalphitanum* and *P. dominula* (Supplementary Notes B6). v2, re-classified. (TIF)

S12 Fig Maximum likelihood analysis of phylogenetic relationships among eukaryotic Dicer homologs from animals, plants, and fungi. *M. amalphitanum* and *T. pretiosum* Dcr-1 and Dcr-2 homologs are denoted by red dots. Multiple alignments of CDS sequences were performed using Muscle v3.8 with default settings. Phylogenetic trees were generated under the maximum likelihood criterion using PhyML 3.0 (GTR model, NNI topological moves and likelihood branch supports). All manipulations of phylogenetic trees were performed using FigTree. Scale bar, nucleotide substitutions per site.
(PNG)

S13 Fig Box plot of percent identity between BLASTN hits for *M. amalphitanum* integrase-related TE transcripts, binned by copy count. High-copy hits represent MITEs. (PNG)

S14 Fig An overview of the missing gene analysis pipeline and its results. (TIF)

S1 Table Paired-end DNA-libraries used for *M. amalphitanum* genome sequencing. (DOCX)

S2 Table *M. amalphitanum* genome assembly statistics using ABySS, SPAdes, CLC and Velvet software (contigs). (DOCX)

S3 Table Evaluation of the *M. amalphitanum* genome and transcriptome assemblies using the BUSCO v3 (benchmarking universal single-copy orthologs) Hymenoptera gene set. (DOCX)

S4 Table *M. amalphitanum* and *C. solmsi* transcriptome assembly statistics using Trinity software (contigs). (DOCX)

S5 Table Reference data sets used for *M. amalphitanum* genome and transcriptome data analysis. (DOCX)

S6 Table Trinotate statistics for *M. amalphitanum*, *C. solmsi, D. alloeum, F. arisanus, C. vestalis, T. pretiosum* transcriptome assemblies. (DOCX)

S7 Table A set of 78 genes (paralogs and homologs) not covered by *M. amalphitanum* reads. (DOCX)

S8 Table Common putative venom constituents in Chalcidoidea parasitoid wasps *M. amalphitanum*, *C. solmsi, M. spermotrophus, T. pretiosum, N. vitripennis*. (DOCX)
