# Supplementary figures and images for: A partial genome assembly of the miniature parasitoid wasp, Megaphragma amalphitanum
Source: PLoS One. 2019 Dec 23;14(12):e0226485. doi: 10.1371/journal.pone.0226485 (PMC6927652; doi:10.1371/journal.pone.0226485)

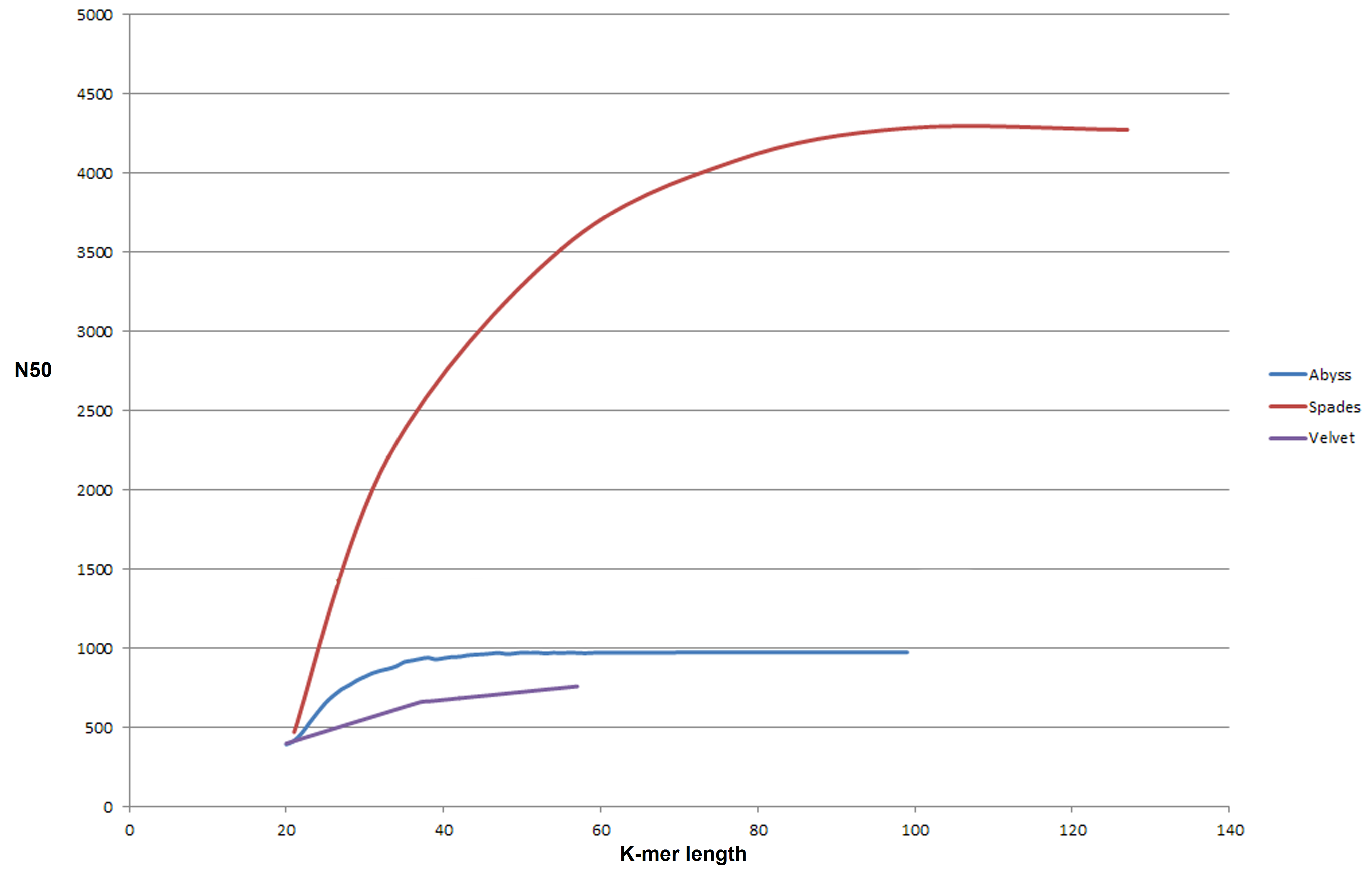

Supplement: S1 Fig — K-mer sizes were matched for ABySS, SPAdes and Velvet. Note: CLC Genomics Workbench does not use k-mer size; CLC assembly was performed with default settings, and the statistics are given in S2 Table. (PNG) [file pone.0226485.s001.png]

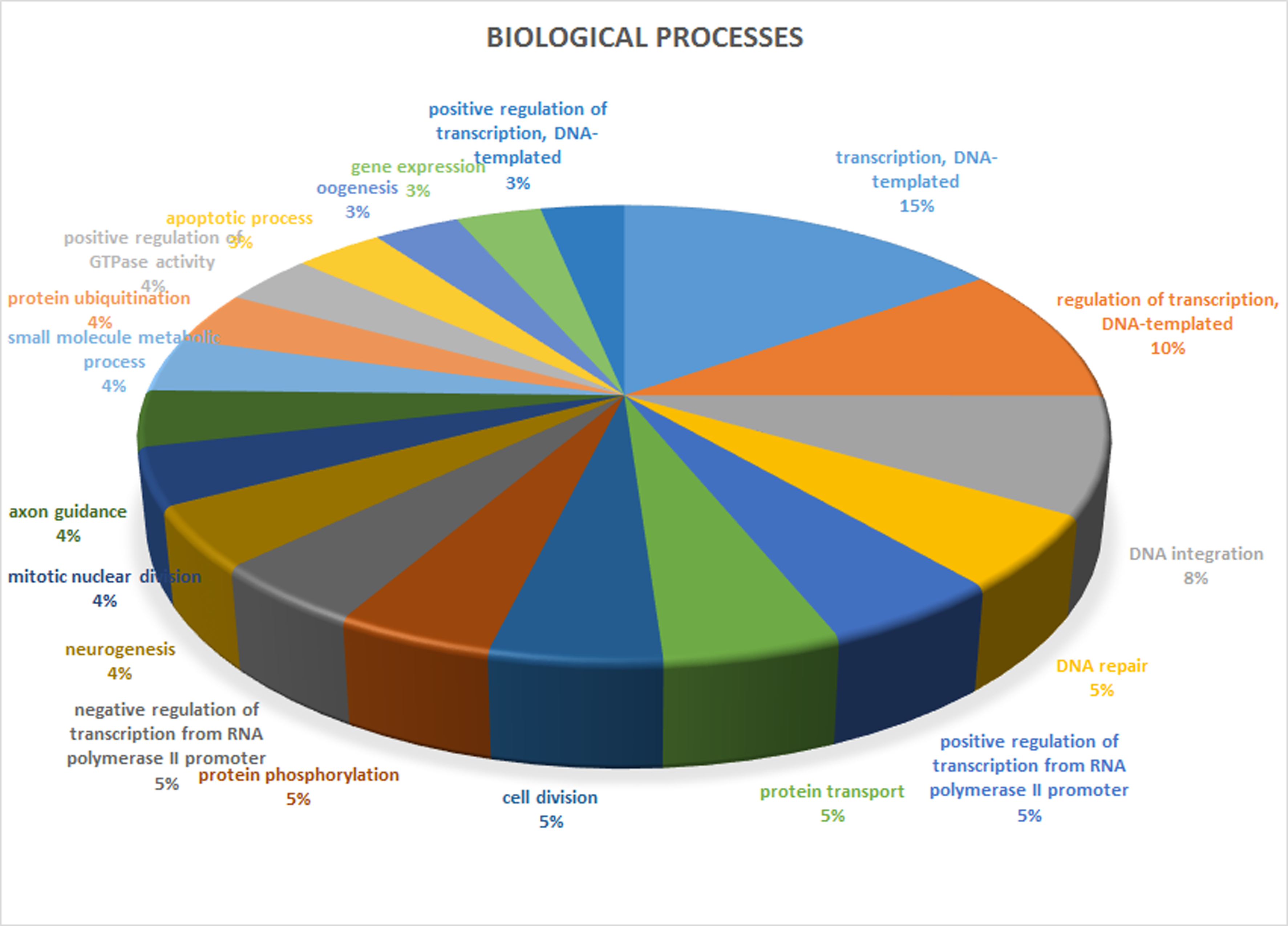

Supplement: S2 Fig — (TIF) [file pone.0226485.s002.tif]

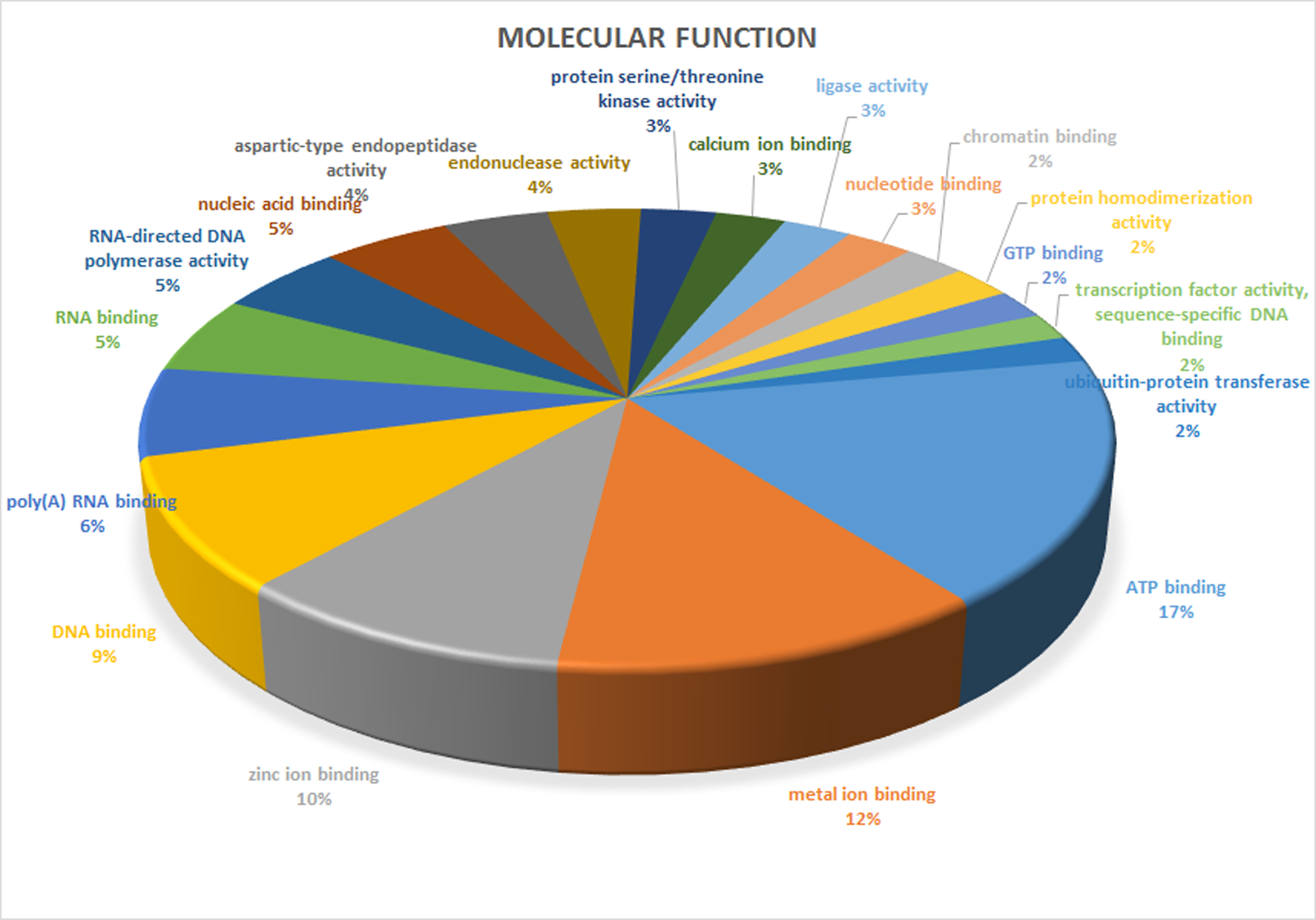

Supplement: S3 Fig — (TIF) [file pone.0226485.s003.tif]

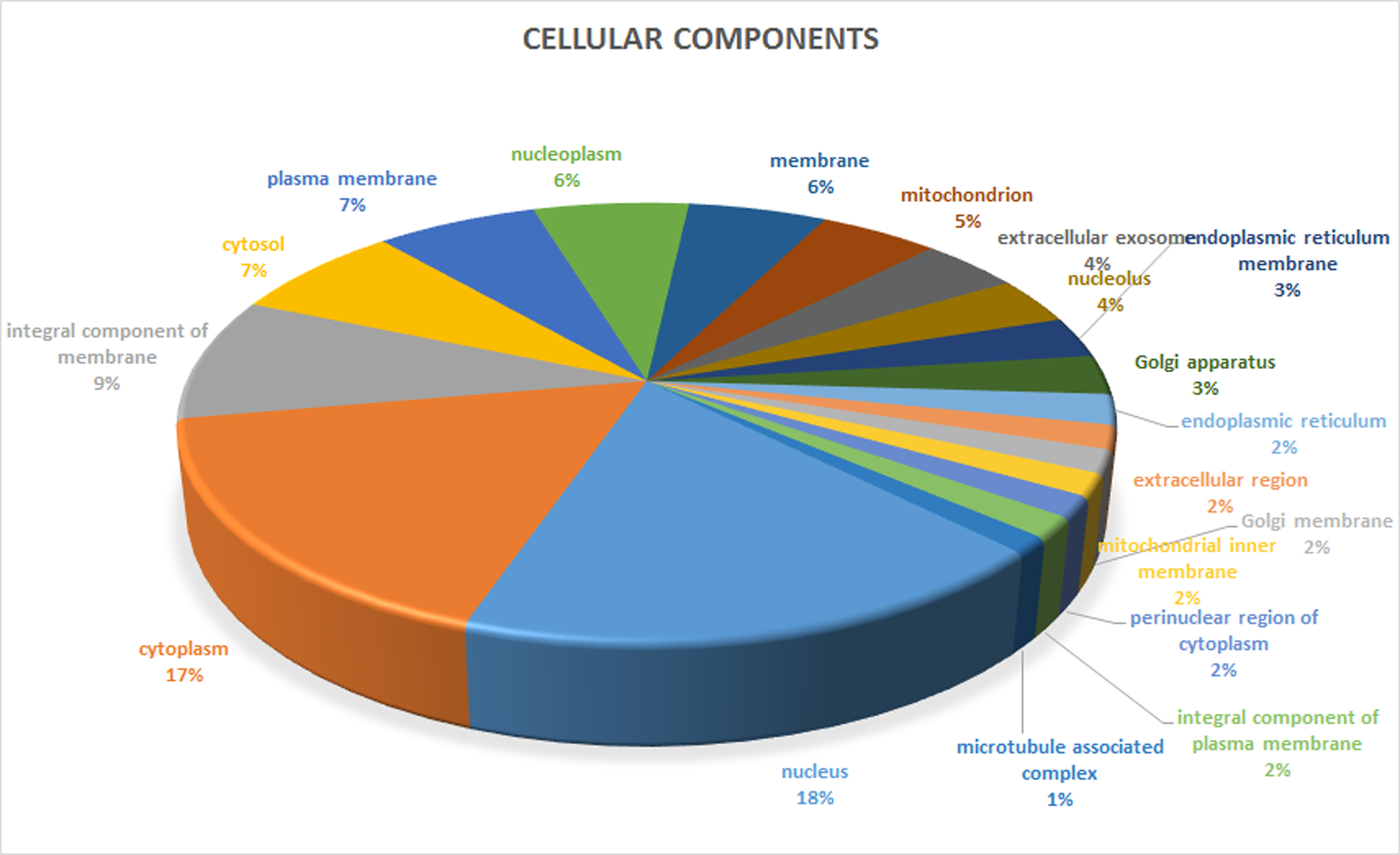

Supplement: S4 Fig — (TIF) [file pone.0226485.s004.tif]

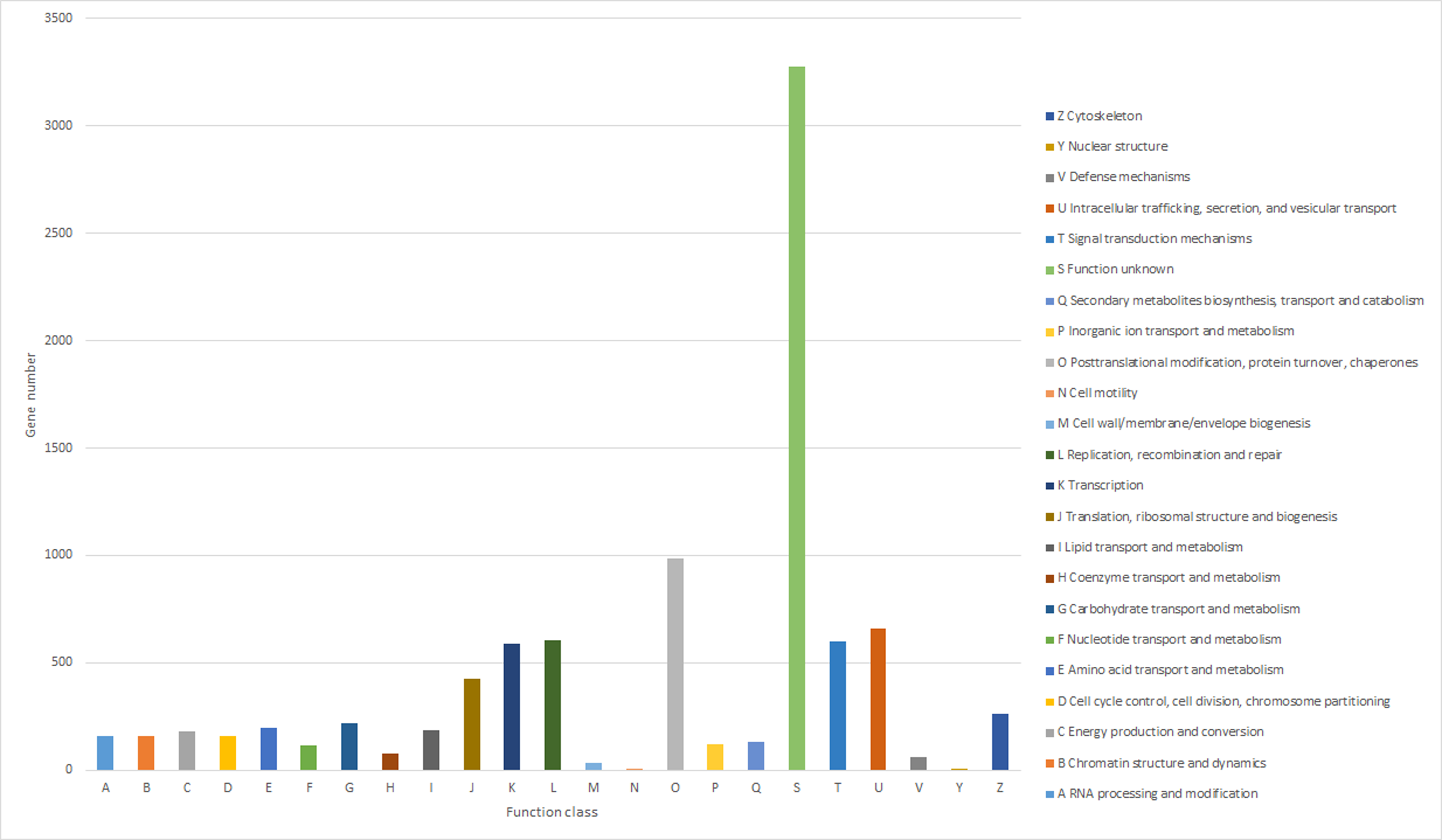

Supplement: S5 Fig — (TIF) [file pone.0226485.s005.tif]

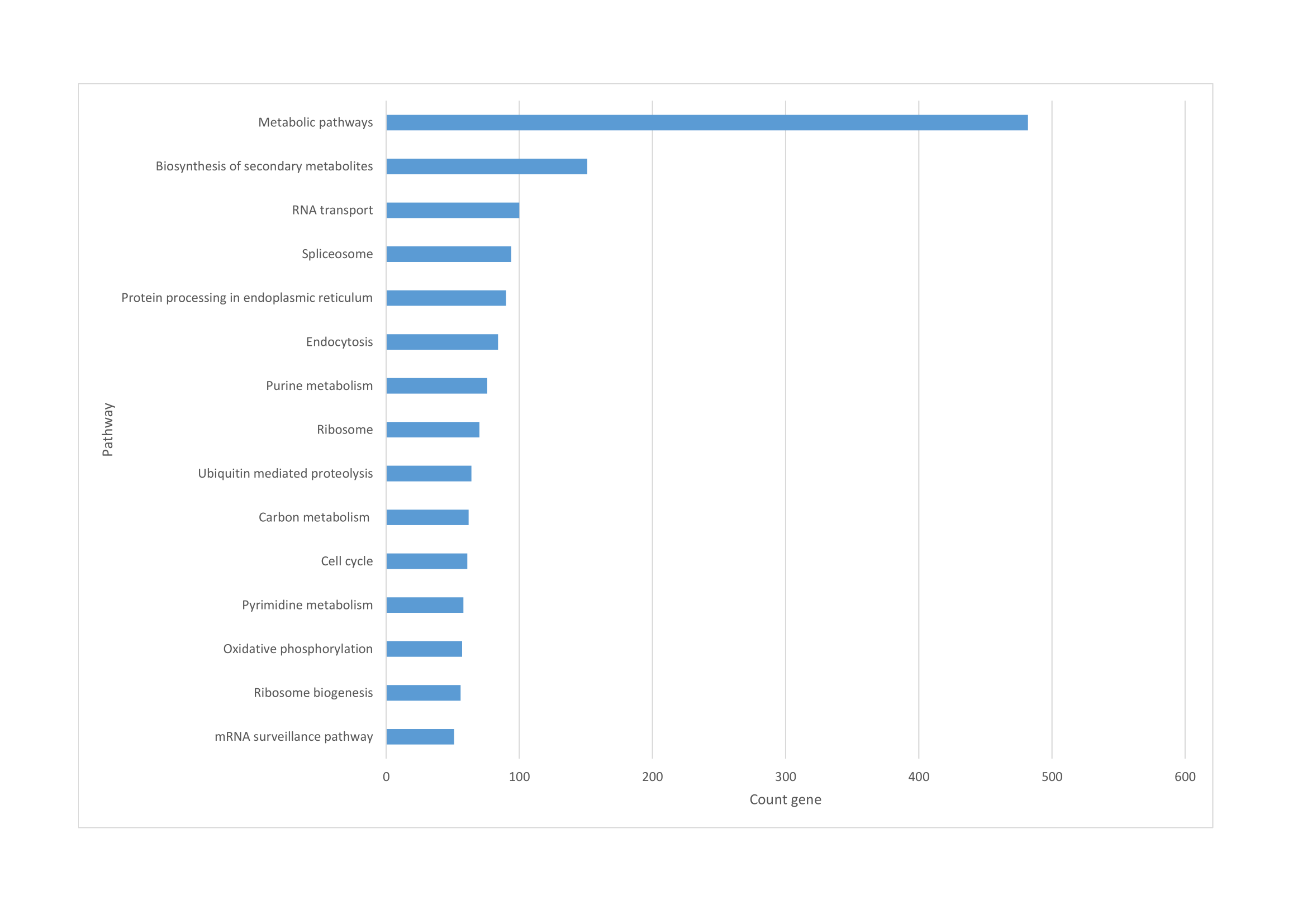

Supplement: S6 Fig — (TIFF) [file pone.0226485.s006.tiff]

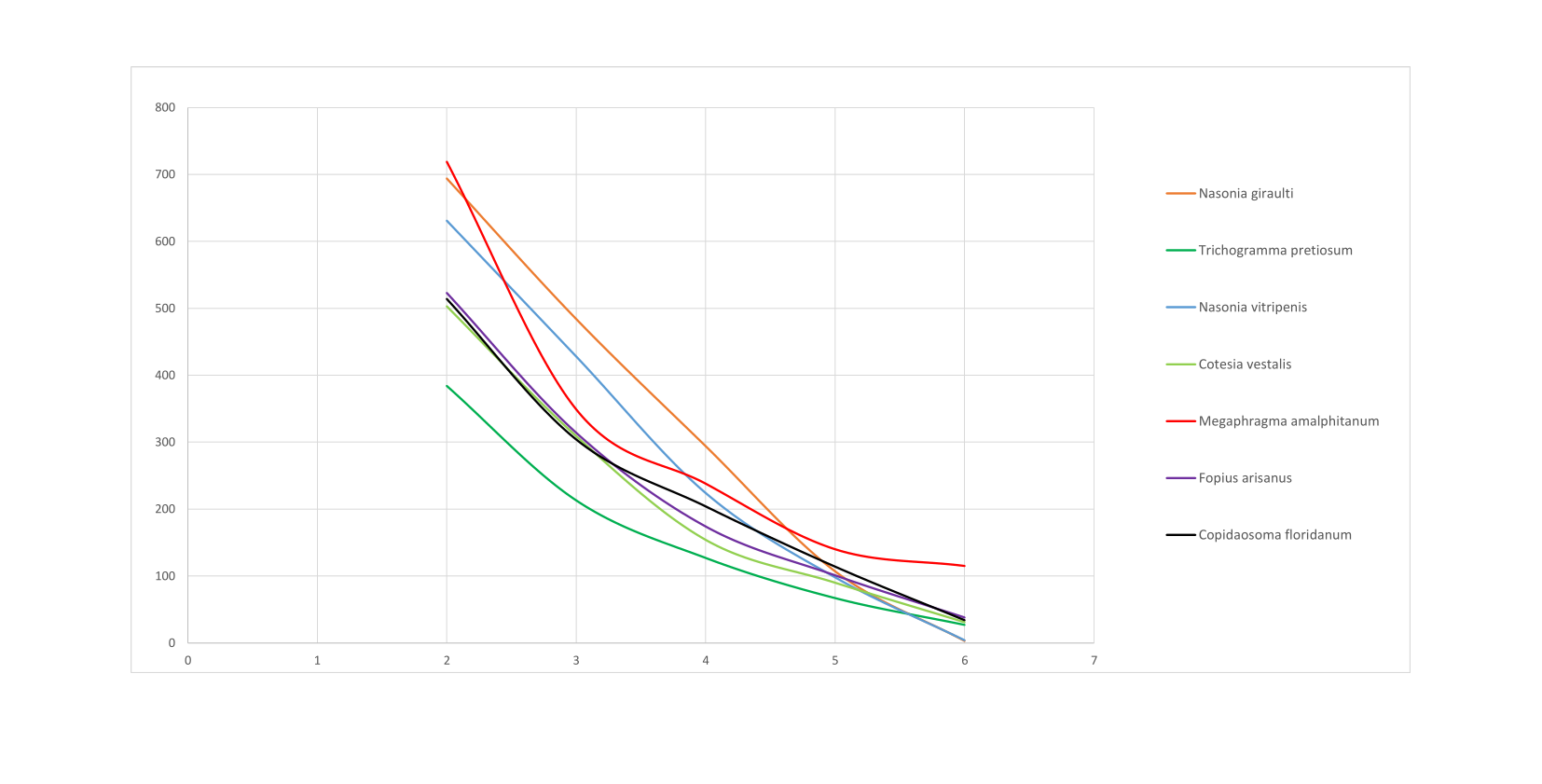

Supplement: S10 Fig — Y-axis: number of genes; X-axis: number of hymenopteran genomes analysed. (TIF) [file pone.0226485.s010.tif]

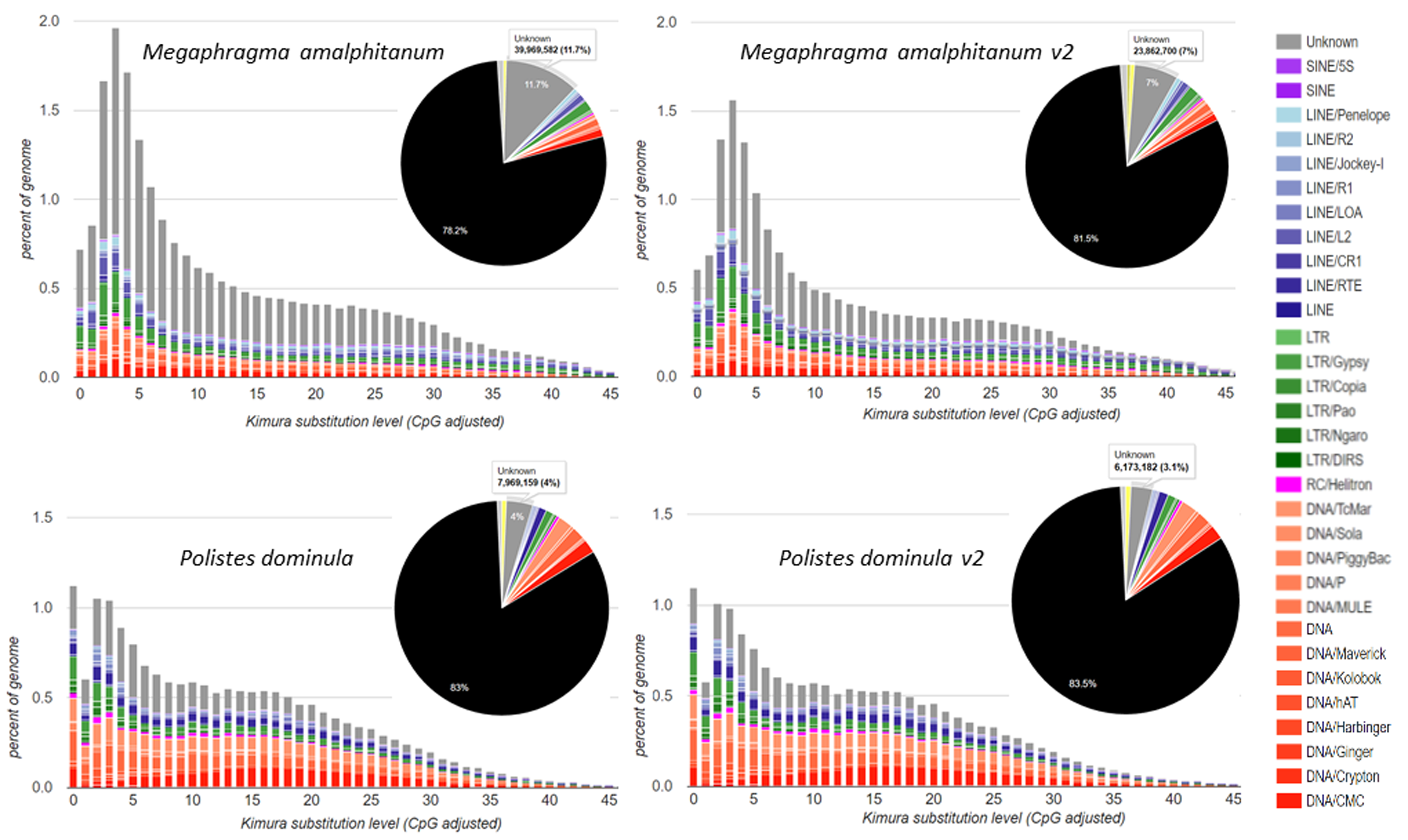

Supplement: S11 Fig — (TIF) [file pone.0226485.s011.tif]

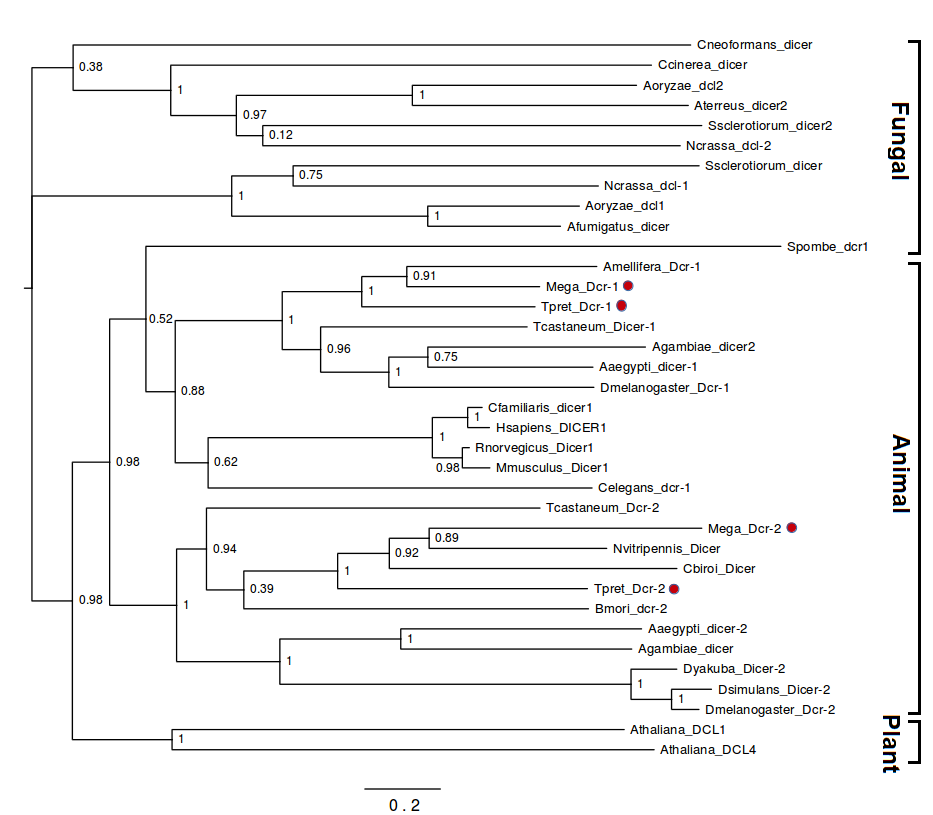

Supplement: S12 Fig — Multiple alignments of CDS sequences were performed using Muscle v3.8 with default settings. Phylogenetic trees were generated under the maximum likelihood criterion using PhyML 3.0 (GTR model, NNI topological moves and likelihood branch supports). All manipulations of phylogenetic trees were performed using FigTree. Scale bar, nucleotide substitutions per site. (PNG) [file pone.0226485.s012.png]

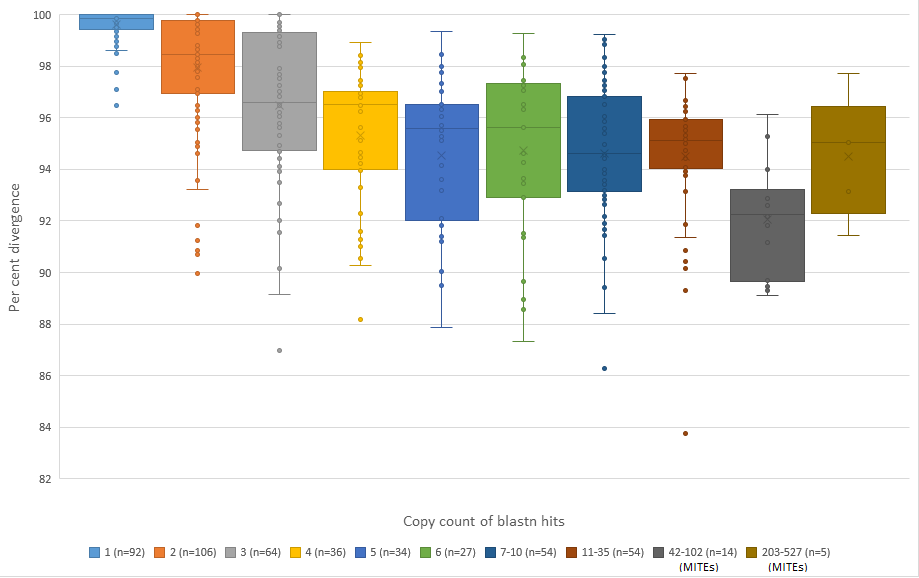

Supplement: S13 Fig — (PNG) [file pone.0226485.s013.png]

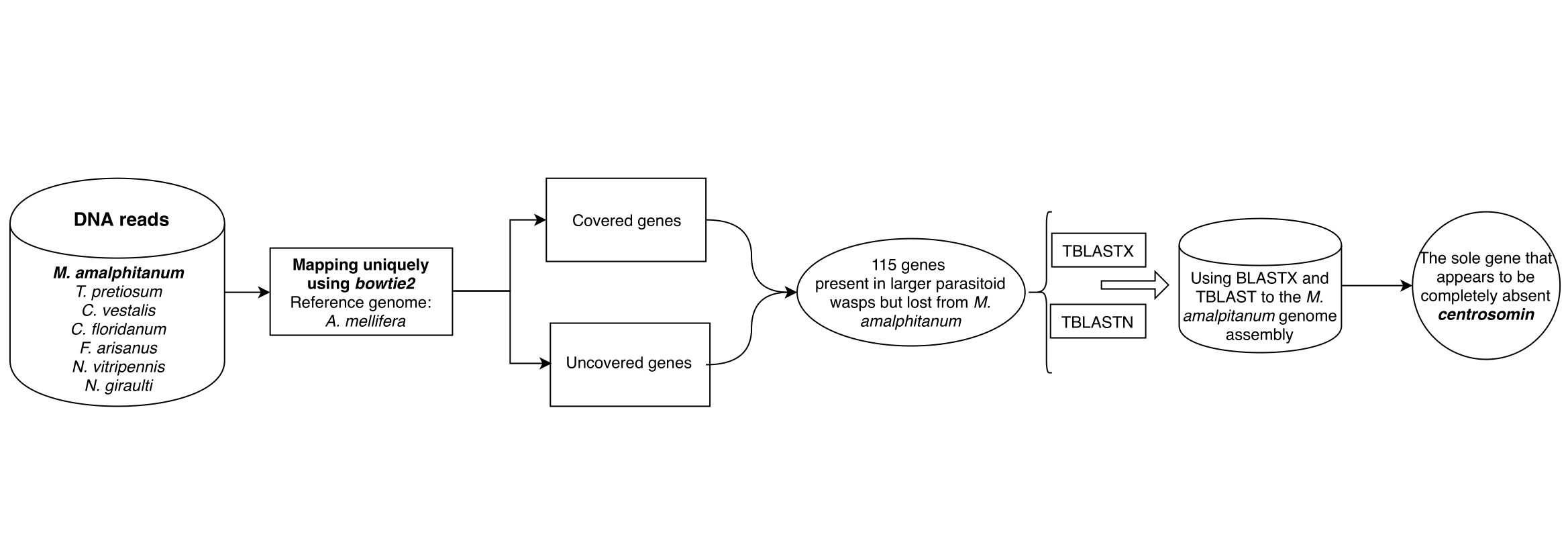

Supplement: S14 Fig — (TIF) [file pone.0226485.s014.tif]
