# Supplementary material for: A partial genome assembly of the miniature parasitoid wasp, Megaphragma amalphitanum
Source: PLoS One. 2019 Dec 23;14(12):e0226485. doi: 10.1371/journal.pone.0226485 (PMC6927652; doi:10.1371/journal.pone.0226485)
Supplement: S1 Table — (DOCX) [file pone.0226485.s015.docx]

S1 Table. Paired-end DNA-libraries used for *M. amalphitanum* genome sequencing.

| **Library name** | **Library concentration, Qubit, ng/µl** | **Average library size, Agilent 2100 Bioanalyzer with a High-Sensitivity DNA chip** | **SRA accession** |
| --- | --- | --- | --- |
| **DNA-library1 – whole insect (ten individuals)** | 1.84 | 350 bp | SRR4340083 |
| **DNA-library2 –**  **body: thorax and abdomen (ten individuals)** | 11.3 | 315 bp | SRR5982987 |
| **DNA-library3 – head (ten individuals)** | 1.8 | 334 bp | SRR5982986 |
