# Supplementary material for: A partial genome assembly of the miniature parasitoid wasp, Megaphragma amalphitanum
Source: PLoS One. 2019 Dec 23;14(12):e0226485. doi: 10.1371/journal.pone.0226485 (PMC6927652; doi:10.1371/journal.pone.0226485)
Supplement: S2 Table — (DOCX) [file pone.0226485.s016.docx]

S2 Table. *M. amalphitanum* genome assembly statistics using ABySS, SPAdes, CLC and Velvet software (contigs).

| **n** | **n:N50** | **N50** | **Maximum contig length, bp** | **Summary assembly size, bp** | ***De novo* assembler** |
| --- | --- | --- | --- | --- | --- |
| **541950** | 31780 | 869 | 19313 | 2.91E+08 | Velvet |
| **484255** | 53248 | 1425 | 33321 | 3.07E+08 | CLC |
| **553157** | 20847 | 4285 | 56202 | 3.58E+08 | SPAdes |
| **4.58E+06** | 60283 | 974 | 21336 | 2.20E+08 | ABySS |
