# Supplementary material for: A partial genome assembly of the miniature parasitoid wasp, Megaphragma amalphitanum
Source: PLoS One. 2019 Dec 23;14(12):e0226485. doi: 10.1371/journal.pone.0226485 (PMC6927652; doi:10.1371/journal.pone.0226485)
Supplement: S3 Table — (DOCX) [file pone.0226485.s017.docx]

S3 Table. Evaluation of the *M. amalphitanum* genome and transcriptome assemblies using the BUSCO v3 (benchmarking universal single-copy orthologs) Hymenoptera gene set.

| ***M. amalphitanum*** | **Complete (%)** | **Duplicated (%)** | **Fragment (%)** | **Missing (%)** |
| --- | --- | --- | --- | --- |
| **Genome** | 80.4% | 1.3% | 9.8% | 9.8% |
| **Transcriptome** | 24.65% | 5.7% | 28.12% | 42.37% |
