# Supplementary material for: A partial genome assembly of the miniature parasitoid wasp, Megaphragma amalphitanum
Source: PLoS One. 2019 Dec 23;14(12):e0226485. doi: 10.1371/journal.pone.0226485 (PMC6927652; doi:10.1371/journal.pone.0226485)
Supplement: S4 Table — (DOCX) [file pone.0226485.s018.docx]

S4 Table. *M. amalphitanum* and *C. solmsi* transcriptome assembly statistics using Trinity software (contigs).

| **N** | **n:N50** | **N50** | **Maximum contig length, bp** | **Summary assembly size, bp** | **Wasp species** |
| --- | --- | --- | --- | --- | --- |
| **46841** | 13109 | 633 | 9503 | 3.74×10^7^ | *M. amalphitanum* |
| **62786** | 12699 | 724 | 15263 | 3.64×10^7^ | *C. solmsi* |
