# Supplementary material for: A partial genome assembly of the miniature parasitoid wasp, Megaphragma amalphitanum
Source: PLoS One. 2019 Dec 23;14(12):e0226485. doi: 10.1371/journal.pone.0226485 (PMC6927652; doi:10.1371/journal.pone.0226485)
Supplement: S5 Table — (DOCX) [file pone.0226485.s019.docx]

S5 Table. Reference data sets used for *M. amalphitanum* genome and transcriptome data analysis.

| **Parasitoid wasp species used in analysis** | **Taxonomy (Suborder, Superfamily, Family)** | **Body size, mm** | **Genome size, Mbp** | **Neuron number in the brain** | **Source link** | **Data type** | **Usage** |
| --- | --- | --- | --- | --- | --- | --- | --- |
| ***Megaphragma amalphitanum*** | Apocrita; Chalcidoidea; Trichogrammatidae | 0.25 | 346 | 4600 | <https://www.ncbi.nlm.nih.gov/bioproject/PRJNA344956> | SRA reads | Transcriptome and Genome  Data Analysis |
| ***Trichogramma pretiosum*** | Apocrita; Chalcidoidea; Trichogrammatidae | 0.5 | 195.1 | 18000 | <https://www.ncbi.nlm.nih.gov/bioproject/275661> | SRA reads | Transcriptome  Data Analysis |
| ***Ceratosolen solmsi*** | Apocrita; Chalcidoidea; Agaonidae | 2.7 | 278 | No data | <http://sra.dnanexus.com/studies/SRP029703/experiments> | SRA reads | Transcriptome  Data Analysis: 62,786 contigs assembled |
| ***Copidosoma floridanum*** | Apocrita; Chalcidoidea; Encyrtidae | 1.2 | 555 | No data | <https://www.ncbi.nlm.nih.gov/sra/SRR947009> <https://www.ncbi.nlm.nih.gov/sra/SRR947010> | SRA reads | Genome Data Analysis |
| ***Nasonia vitripennis*** | Apocrita; Chalcidoidea; Pteromalidae | 2.2 | 295.8 | No data | <https://www.ncbi.nlm.nih.gov/assembly/GCF_000002325.3> | Genome | Genome Data Analysis |
| ***Nasonia giraulti*** | Apocrita; Chalcidoidea; Pteromalidae | 2.3 | 283.6 | No data | <https://www.ncbi.nlm.nih.gov/assembly/GCA_000004775.1> | Genome | Genome Data Analysis |
| ***Diachasma alloeum*** | Apocrita; Ichneumonoidea; Braconidae | 4.2 | 388.7 | No data | <https://www.ncbi.nlm.nih.gov/nuccore/GECN00000000.1> | Complete  transcriptome | Transcriptome  Data Analysis: 131,607 contigs; 112,635,971 bp total |
| ***Fopius arisanus*** | Apocrita; Ichneumonoidea; Braconidae | 4.5 | 153,6 | No data | <https://www.ncbi.nlm.nih.gov/sra/SRR1560675>  <https://www.ncbi.nlm.nih.gov/sra/SRR1560673>  <https://www.ncbi.nlm.nih.gov/sra/SRR1560668>  <https://www.ncbi.nlm.nih.gov/nuccore/748430103> | Complete transcriptome and SRA reads | Genome Data Analysis. Transcriptome  Data Analysis15,346 contigs; 50,620,881 bp total |
| ***Cotesia vestalis*** | Apocrita; Ichneumonoidea; Braconidae | 1.9 | 131,9 | No data | <https://www.ncbi.nlm.nih.gov/sra/SRR2029645>  <https://www.ncbi.nlm.nih.gov/nuccore/511518236> | Complete  transcriptome | Genome Data Analysis. Transcriptome  Data Analysis: 30,024 contigs; 27,114,579 bp total; teratocyte (extraembryonic cell) |
| ***Megastigmus spermotrophus*** | Apocrita; Chalcidoidea; Torymidae | 2.8 | No data | No data | [https://www.ncbi.nlm.nih.gov//bioproject/PRJNA274192](https://www.ncbi.nlm.nih.gov/bioproject/PRJNA274192) | SRA reads | Transcriptome  Data Analysis |
| **Reference Hymenoptera species** | - | - | - | - |  |  |  |
| ***Apis mellifera*** | Apocrita; Apoidea; Apidae | 15 | 246.9 | 850000 – 1200000 | <http://metazoa.ensembl.org/Apis_mellifera/Info/Index> | Complete genome | Genome and transcriptome analysis |
