# Supplementary material for: A partial genome assembly of the miniature parasitoid wasp, Megaphragma amalphitanum
Source: PLoS One. 2019 Dec 23;14(12):e0226485. doi: 10.1371/journal.pone.0226485 (PMC6927652; doi:10.1371/journal.pone.0226485)
Supplement: S6 Table — (DOCX) [file pone.0226485.s020.docx]

S6 Table. Trinotate statistics for *M. amalphitanum, C. solmsi*, *D. alloeum*, *F. arisanus*, *C. vestalis*, *T. pretiosum* transcriptome assemblies.

| **Parasitoid wasp species** | **Number of transcripts used for annotation** | **Number of transcripts annotated by BLASTX** | **Gene ontology for BLASTX data** | **Number of transcripts annotated by EggNog database** | **Number of transcripts annotated by KEGG**  **database** | **Number of**  **transcripts**  **annotated by BLASTP** | **Number of transcripts annotated by Pfam** | **Gene ontology for**  **Pfam** | **Prediction of transmembrane helices in proteins (TmHMM)** |
| --- | --- | --- | --- | --- | --- | --- | --- | --- | --- |
| ***C. solmsi*** | 63783 | 17816 | 17046 | 14833 | 14499 | 12592 | 10826 | 7136 | 2159 |
| ***D. alloeum*** | 135999 | 42492 | 39607 | 27183 | 33384 | 29569 | 28427 | 19335 | 6840 |
| ***F. arisanus*** | 22452 | 19290 | 16792 | 14685 | 14622 | 16843 | 16379 | 16792 | 4509 |
| ***C. vestalis*** | 31395 | 12693 | 11994 | 10537 | 10383 | 10578 | 9768 | 6421 | 2074 |
| ***T. pretiosum*** | 20818 | 17351 | 15802 | 13736 | 13609 | 15891 | 16040 | 11801 | 4308 |
| ***M. amalphitanum*** | 46841 | 12238 | 10721 | 8810 | 6130 | 8193 | 6808 | 4196 | 1197 |
