# Supplementary material for: A partial genome assembly of the miniature parasitoid wasp, Megaphragma amalphitanum
Source: PLoS One. 2019 Dec 23;14(12):e0226485. doi: 10.1371/journal.pone.0226485 (PMC6927652; doi:10.1371/journal.pone.0226485)
Supplement: S7 Table — (DOCX) [file pone.0226485.s021.docx]

S7 Table. A set of 78 genes (paralogs and homologs) not covered by *M. amalphitanum* reads.

| ***A. melifera***  **gene ID** | ***Drosophila melanogaster* gene ID** | ***D. melanogaster* homologous gene** |
| --- | --- | --- |
| [**GB45679**](http://metazoa.ensembl.org/apis_mellifera/Gene/Summary?db=core;g=GB45679) | [FBgn0261823](http://metazoa.ensembl.org/drosophila_melanogaster/Gene/Summary?g=FBgn0261823) | Additional sex combs [Source:FlyBase;Acc:FBgn0261823] |
| [**GB47271**](http://metazoa.ensembl.org/apis_mellifera/Gene/Summary?db=core;g=GB47271) | [FBgn0022710](http://metazoa.ensembl.org/drosophila_melanogaster/Gene/Summary?g=FBgn0022710) | Adenylyl cyclase 35C [Source:FlyBase;Acc:FBgn0022710] |
| **[GB53936](http://metazoa.ensembl.org/apis_mellifera/Gene/Summary?db=core;g=GB53936)** | [FBgn0000228](http://metazoa.ensembl.org/drosophila_melanogaster/Gene/Summary?g=FBgn0000228) | Blastoderm-specific gene 25D [Source:FlyBase;Acc:FBgn0000228] |
| [**GB42838**](http://metazoa.ensembl.org/apis_mellifera/Gene/Summary?db=core;g=GB42838) | [FBgn0031883](http://metazoa.ensembl.org/drosophila_melanogaster/Gene/Summary?g=FBgn0031883) | Caper [Source:FlyBase;Acc:FBgn0031883] |
| [**GB45937**](http://metazoa.ensembl.org/apis_mellifera/Gene/Summary?db=core;g=GB45937) | [FBgn0013765](http://metazoa.ensembl.org/drosophila_melanogaster/Gene/Summary?g=FBgn0013765) | centrosomin [Source:FlyBase;Acc:FBgn0013765] |
| [**GB44187**](http://metazoa.ensembl.org/apis_mellifera/Gene/Summary?db=core;g=GB44187) | [FBgn0028387](http://metazoa.ensembl.org/drosophila_melanogaster/Gene/Summary?g=FBgn0028387) | chameau [Source:FlyBase;Acc:FBgn0028387] |
| [**GB44210**](http://metazoa.ensembl.org/apis_mellifera/Gene/Summary?db=core;g=GB44210) | [FBgn0037240](http://metazoa.ensembl.org/drosophila_melanogaster/Gene/Summary?g=FBgn0037240) | Contactin [Source:FlyBase;Acc:FBgn0037240] |
| [**GB41346**](http://metazoa.ensembl.org/apis_mellifera/Gene/Summary?db=core;g=GB41346) | [FBgn0041342](http://metazoa.ensembl.org/drosophila_melanogaster/Gene/Summary?g=FBgn0041342) | CTP:phosphocholine cytidylyltransferase 1 [Source:FlyBase;Acc:FBgn0041342] |
| [**GB41346**](http://metazoa.ensembl.org/apis_mellifera/Gene/Summary?db=core;g=GB41346) | [FBgn0035231](http://metazoa.ensembl.org/drosophila_melanogaster/Gene/Summary?g=FBgn0035231) | CTP:phosphocholine cytidylyltransferase 2 [Source:FlyBase;Acc:FBgn0035231] |
| [**GB46429**](http://metazoa.ensembl.org/apis_mellifera/Gene/Summary?db=core;g=GB46429) | [FBgn0000527](http://metazoa.ensembl.org/drosophila_melanogaster/Gene/Summary?g=FBgn0000527) | ebony [Source:FlyBase;Acc:FBgn0000527] |
| [**GB50237**](http://metazoa.ensembl.org/apis_mellifera/Gene/Summary?db=core;g=GB50237) | [FBgn0033354](http://metazoa.ensembl.org/drosophila_melanogaster/Gene/Summary?g=FBgn0033354) | Fanconi anemia complementation group I homologue [Source:FlyBase;Acc:FBgn0033354] |
| [**GB45272**](http://metazoa.ensembl.org/apis_mellifera/Gene/Summary?db=core;g=GB45272) | [FBgn0001987](http://metazoa.ensembl.org/drosophila_melanogaster/Gene/Summary?g=FBgn0001987) | Gliotactin [Source:FlyBase;Acc:FBgn0001987] |
| [**GB55822**](http://metazoa.ensembl.org/apis_mellifera/Gene/Summary?db=core;g=GB55822) | [FBgn0266136](http://metazoa.ensembl.org/drosophila_melanogaster/Gene/Summary?g=FBgn0266136) | Guanylyl cyclase at 76C [Source:FlyBase;Acc:FBgn0266136] |
| [**GB42147**](http://metazoa.ensembl.org/apis_mellifera/Gene/Summary?db=core;g=GB42147) | [FBgn0030600](http://metazoa.ensembl.org/drosophila_melanogaster/Gene/Summary?g=FBgn0030600) | highwire [Source:FlyBase;Acc:FBgn0030600] |
| [**GB44850**](http://metazoa.ensembl.org/apis_mellifera/Gene/Summary?db=core;g=GB44850) | [FBgn0005654](http://metazoa.ensembl.org/drosophila_melanogaster/Gene/Summary?g=FBgn0005654) | latheo [Source:FlyBase;Acc:FBgn0005654] |
| [**GB43279**](http://metazoa.ensembl.org/apis_mellifera/Gene/Summary?db=core;g=GB43279) | [FBgn0034282](http://metazoa.ensembl.org/drosophila_melanogaster/Gene/Summary?g=FBgn0034282) | Mapmodulin [Source:FlyBase;Acc:FBgn0034282] |
| [**GB43420**](http://metazoa.ensembl.org/apis_mellifera/Gene/Summary?db=core;g=GB43420) | [FBgn0261109](http://metazoa.ensembl.org/drosophila_melanogaster/Gene/Summary?g=FBgn0261109) | marionette [Source:FlyBase;Acc:FBgn0261109] |
| [**GB52997**](http://metazoa.ensembl.org/apis_mellifera/Gene/Summary?db=core;g=GB52997) | [FBgn0265988](http://metazoa.ensembl.org/drosophila_melanogaster/Gene/Summary?g=FBgn0265988) | mauve [Source:FlyBase;Acc:FBgn0265988] |
| [**GB44910**](http://metazoa.ensembl.org/apis_mellifera/Gene/Summary?db=core;g=GB44910) | [FBgn0027497](http://metazoa.ensembl.org/drosophila_melanogaster/Gene/Summary?g=FBgn0027497) | MLF1-adaptor molecule [Source:FlyBase;Acc:FBgn0027497] |
| [**GB55787**](http://metazoa.ensembl.org/apis_mellifera/Gene/Summary?db=core;g=GB55787) | [FBgn0002878](http://metazoa.ensembl.org/drosophila_melanogaster/Gene/Summary?g=FBgn0002878) | mutagen-sensitive 101 [Source:FlyBase;Acc:FBgn0002878] |
| [**GB49559**](http://metazoa.ensembl.org/apis_mellifera/Gene/Summary?db=core;g=GB49559) | [FBgn0016919](http://metazoa.ensembl.org/drosophila_melanogaster/Gene/Summary?g=FBgn0016919) | no mechanoreceptor potential B [Source:FlyBase;Acc:FBgn0016919] |
| [**GB43945**](http://metazoa.ensembl.org/apis_mellifera/Gene/Summary?db=core;g=GB43945) | [FBgn0061200](http://metazoa.ensembl.org/drosophila_melanogaster/Gene/Summary?g=FBgn0061200) | Nucleoporin 153kD [Source:FlyBase;Acc:FBgn0061200] |
| [**GB43591**](http://metazoa.ensembl.org/apis_mellifera/Gene/Summary?db=core;g=GB43591) | [FBgn0026058](http://metazoa.ensembl.org/drosophila_melanogaster/Gene/Summary?g=FBgn0026058) | Ods-site homeobox [Source:FlyBase;Acc:FBgn0026058] |
| [**GB41452**](http://metazoa.ensembl.org/apis_mellifera/Gene/Summary?db=core;g=GB41452) | [FBgn0023517](http://metazoa.ensembl.org/drosophila_melanogaster/Gene/Summary?g=FBgn0023517) | Phosphoglycerate mutase 5 [Source:FlyBase;Acc:FBgn0023517] |
| [**GB41452**](http://metazoa.ensembl.org/apis_mellifera/Gene/Summary?db=core;g=GB41452) | [FBgn0035004](http://metazoa.ensembl.org/drosophila_melanogaster/Gene/Summary?g=FBgn0035004) | Phosphoglycerate mutase 5-2 [Source:FlyBase;Acc:FBgn0035004] |
| [**GB46511**](http://metazoa.ensembl.org/apis_mellifera/Gene/Summary?db=core;g=GB46511) | [FBgn0035405](http://metazoa.ensembl.org/drosophila_melanogaster/Gene/Summary?g=FBgn0035405) | piefke [Source:FlyBase;Acc:FBgn0035405] |
| [**GB46270**](http://metazoa.ensembl.org/apis_mellifera/Gene/Summary?db=core;g=GB46270) | [FBgn0025740](http://metazoa.ensembl.org/drosophila_melanogaster/Gene/Summary?g=FBgn0025740) | Plexin B [Source:FlyBase;Acc:FBgn0025740] |
| [**GB54796**](http://metazoa.ensembl.org/apis_mellifera/Gene/Summary?db=core;g=GB54796) | [FBgn0025334](http://metazoa.ensembl.org/drosophila_melanogaster/Gene/Summary?g=FBgn0025334) | Putative homeodomain protein [Source:FlyBase;Acc:FBgn0025334] |
| [**GB41985**](http://metazoa.ensembl.org/apis_mellifera/Gene/Summary?db=core;g=GB41985) | [FBgn0024941](http://metazoa.ensembl.org/drosophila_melanogaster/Gene/Summary?g=FBgn0024941) | Regulator of G-protein signalling 7 [Source:FlyBase;Acc:FBgn0024941] |
| [**GB53284**](http://metazoa.ensembl.org/apis_mellifera/Gene/Summary?db=core;g=GB53284) | [FBgn0011829](http://metazoa.ensembl.org/drosophila_melanogaster/Gene/Summary?g=FBgn0011829) | Ret oncogene [Source:FlyBase;Acc:FBgn0011829] |
| [**GB42110**](http://metazoa.ensembl.org/apis_mellifera/Gene/Summary?db=core;g=GB42110) | [FBgn0264087](http://metazoa.ensembl.org/drosophila_melanogaster/Gene/Summary?g=FBgn0264087) | Slowpoke binding protein [Source:FlyBase;Acc:FBgn0264087] |
| [**GB44534**](http://metazoa.ensembl.org/apis_mellifera/Gene/Summary?db=core;g=GB44534) | [FBgn0039141](http://metazoa.ensembl.org/drosophila_melanogaster/Gene/Summary?g=FBgn0039141) | spastin [Source:FlyBase;Acc:FBgn0039141] |
| [**GB43591**](http://metazoa.ensembl.org/apis_mellifera/Gene/Summary?db=core;g=GB43591) | [FBgn0024184](http://metazoa.ensembl.org/drosophila_melanogaster/Gene/Summary?g=FBgn0024184) | unc-4 [Source:FlyBase;Acc:FBgn0024184] |
| [**GB40007**](http://metazoa.ensembl.org/apis_mellifera/Gene/Summary?db=core;g=GB40007) |  |  |
| [**GB40447**](http://metazoa.ensembl.org/apis_mellifera/Gene/Summary?db=core;g=GB40447) |  |  |
| [**GB40540**](http://metazoa.ensembl.org/apis_mellifera/Gene/Summary?db=core;g=GB40540) | [FBgn0033916](http://metazoa.ensembl.org/drosophila_melanogaster/Gene/Summary?g=FBgn0033916) |  |
| [**GB41035**](http://metazoa.ensembl.org/apis_mellifera/Gene/Summary?db=core;g=GB41035) | [FBgn0050421](http://metazoa.ensembl.org/drosophila_melanogaster/Gene/Summary?g=FBgn0050421) |  |
| [**GB41249**](http://metazoa.ensembl.org/apis_mellifera/Gene/Summary?db=core;g=GB41249) |  |  |
| [**GB41330**](http://metazoa.ensembl.org/apis_mellifera/Gene/Summary?db=core;g=GB41330) |  | 26S proteasome complex subunit DSS1 |
| [**GB41486**](http://metazoa.ensembl.org/apis_mellifera/Gene/Summary?db=core;g=GB41486) | [FBgn0035421](http://metazoa.ensembl.org/drosophila_melanogaster/Gene/Summary?g=FBgn0035421) |  |
| [**GB42383**](http://metazoa.ensembl.org/apis_mellifera/Gene/Summary?db=core;g=GB42383) |  |  |
| [**GB44289**](http://metazoa.ensembl.org/apis_mellifera/Gene/Summary?db=core;g=GB44289) |  |  |
| [**GB44766**](http://metazoa.ensembl.org/apis_mellifera/Gene/Summary?db=core;g=GB44766) |  |  |
| **[GB45063](http://metazoa.ensembl.org/apis_mellifera/Gene/Summary?db=core;g=GB45063)** |  | LIM/homeobox Lhx9-like |
| [**GB45314**](http://metazoa.ensembl.org/apis_mellifera/Gene/Summary?db=core;g=GB45314) |  |  |
| [**GB45456**](http://metazoa.ensembl.org/apis_mellifera/Gene/Summary?db=core;g=GB45456) |  |  |
| [**GB45501**](http://metazoa.ensembl.org/apis_mellifera/Gene/Summary?db=core;g=GB45501) |  |  |
| [**GB46013**](http://metazoa.ensembl.org/apis_mellifera/Gene/Summary?db=core;g=GB46013) | [FBgn0032010](http://metazoa.ensembl.org/drosophila_melanogaster/Gene/Summary?g=FBgn0032010) | Mucin-1-like/Nucleoporin NSP1-like |
| [**GB46267**](http://metazoa.ensembl.org/apis_mellifera/Gene/Summary?db=core;g=GB46267) |  |  |
| [**GB46273**](http://metazoa.ensembl.org/apis_mellifera/Gene/Summary?db=core;g=GB46273) |  |  |
| [**GB46668**](http://metazoa.ensembl.org/apis_mellifera/Gene/Summary?db=core;g=GB46668) |  |  |
| [**GB46705**](http://metazoa.ensembl.org/apis_mellifera/Gene/Summary?db=core;g=GB46705) |  |  |
| [**GB46908**](http://metazoa.ensembl.org/apis_mellifera/Gene/Summary?db=core;g=GB46908) | [FBgn0261550](http://metazoa.ensembl.org/drosophila_melanogaster/Gene/Summary?g=FBgn0261550) |  |
| [**GB47425**](http://metazoa.ensembl.org/apis_mellifera/Gene/Summary?db=core;g=GB47425) | [FBgn0037525](http://metazoa.ensembl.org/drosophila_melanogaster/Gene/Summary?g=FBgn0037525) |  |
| [**GB47871**](http://metazoa.ensembl.org/apis_mellifera/Gene/Summary?db=core;g=GB47871) |  |  |
| [**GB47927**](http://metazoa.ensembl.org/apis_mellifera/Gene/Summary?db=core;g=GB47927) |  |  |
| [**GB48315**](http://metazoa.ensembl.org/apis_mellifera/Gene/Summary?db=core;g=GB48315) |  |  |
| [**GB48563**](http://metazoa.ensembl.org/apis_mellifera/Gene/Summary?db=core;g=GB48563) | [FBgn0037094](http://metazoa.ensembl.org/drosophila_melanogaster/Gene/Summary?g=FBgn0037094) |  |
| [**GB48783**](http://metazoa.ensembl.org/apis_mellifera/Gene/Summary?db=core;g=GB48783) |  |  |
| [**GB48948**](http://metazoa.ensembl.org/apis_mellifera/Gene/Summary?db=core;g=GB48948) | [FBgn0032648](http://metazoa.ensembl.org/drosophila_melanogaster/Gene/Summary?g=FBgn0032648) |  |
| [**GB48948**](http://metazoa.ensembl.org/apis_mellifera/Gene/Summary?db=core;g=GB48948) | [FBgn0032647](http://metazoa.ensembl.org/drosophila_melanogaster/Gene/Summary?g=FBgn0032647) |  |
| [**GB48948**](http://metazoa.ensembl.org/apis_mellifera/Gene/Summary?db=core;g=GB48948) | [FBgn0032649](http://metazoa.ensembl.org/drosophila_melanogaster/Gene/Summary?g=FBgn0032649) |  |
| [**GB49002**](http://metazoa.ensembl.org/apis_mellifera/Gene/Summary?db=core;g=GB49002) |  |  |
| [**GB49164**](http://metazoa.ensembl.org/apis_mellifera/Gene/Summary?db=core;g=GB49164) |  |  |
| [**GB49318**](http://metazoa.ensembl.org/apis_mellifera/Gene/Summary?db=core;g=GB49318) |  |  |
| [**GB49375**](http://metazoa.ensembl.org/apis_mellifera/Gene/Summary?db=core;g=GB49375) |  |  |
| [**GB49611**](http://metazoa.ensembl.org/apis_mellifera/Gene/Summary?db=core;g=GB49611) | [FBgn0030058](http://metazoa.ensembl.org/drosophila_melanogaster/Gene/Summary?g=FBgn0030058) |  |
| [**GB50282**](http://metazoa.ensembl.org/apis_mellifera/Gene/Summary?db=core;g=GB50282) | [FBgn0036727](http://metazoa.ensembl.org/drosophila_melanogaster/Gene/Summary?g=FBgn0036727) |  |
| [**GB50282**](http://metazoa.ensembl.org/apis_mellifera/Gene/Summary?db=core;g=GB50282) | [FBgn0029733](http://metazoa.ensembl.org/drosophila_melanogaster/Gene/Summary?g=FBgn0029733) |  |
| [**GB50282**](http://metazoa.ensembl.org/apis_mellifera/Gene/Summary?db=core;g=GB50282) | [FBgn0039840](http://metazoa.ensembl.org/drosophila_melanogaster/Gene/Summary?g=FBgn0039840) |  |
| [**GB50813**](http://metazoa.ensembl.org/apis_mellifera/Gene/Summary?db=core;g=GB50813) |  |  |
| [**GB51260**](http://metazoa.ensembl.org/apis_mellifera/Gene/Summary?db=core;g=GB51260) |  |  |
| [**GB51273**](http://metazoa.ensembl.org/apis_mellifera/Gene/Summary?db=core;g=GB51273) | [FBgn0037949](http://metazoa.ensembl.org/drosophila_melanogaster/Gene/Summary?g=FBgn0037949) |  |
| [**GB51367**](http://metazoa.ensembl.org/apis_mellifera/Gene/Summary?db=core;g=GB51367) |  |  |
| [**GB51482**](http://metazoa.ensembl.org/apis_mellifera/Gene/Summary?db=core;g=GB51482) |  |  |
| [**GB51556**](http://metazoa.ensembl.org/apis_mellifera/Gene/Summary?db=core;g=GB51556) | [FBgn0259224](http://metazoa.ensembl.org/drosophila_melanogaster/Gene/Summary?g=FBgn0259224) |  |
| [**GB51604**](http://metazoa.ensembl.org/apis_mellifera/Gene/Summary?db=core;g=GB51604) |  | Rx1 retinal homeobox |
| [**GB52413**](http://metazoa.ensembl.org/apis_mellifera/Gene/Summary?db=core;g=GB52413) |  |  |
| [**GB53272**](http://metazoa.ensembl.org/apis_mellifera/Gene/Summary?db=core;g=GB53272) |  |  |
| [**GB53972**](http://metazoa.ensembl.org/apis_mellifera/Gene/Summary?db=core;g=GB53972) |  |  |
| [**GB54235**](http://metazoa.ensembl.org/apis_mellifera/Gene/Summary?db=core;g=GB54235) |  |  |
| [**GB54927**](http://metazoa.ensembl.org/apis_mellifera/Gene/Summary?db=core;g=GB54927) | [FBgn0038686](http://metazoa.ensembl.org/drosophila_melanogaster/Gene/Summary?g=FBgn0038686) |  |
| [**GB55148**](http://metazoa.ensembl.org/apis_mellifera/Gene/Summary?db=core;g=GB55148) | [FBgn0035688](http://metazoa.ensembl.org/drosophila_melanogaster/Gene/Summary?g=FBgn0035688) |  |
| [**GB55764**](http://metazoa.ensembl.org/apis_mellifera/Gene/Summary?db=core;g=GB55764) |  |  |
| [**GB55767**](http://metazoa.ensembl.org/apis_mellifera/Gene/Summary?db=core;g=GB55767) |  |  |
| [**GB55822**](http://metazoa.ensembl.org/apis_mellifera/Gene/Summary?db=core;g=GB55822) | [FBgn0261360](http://metazoa.ensembl.org/drosophila_melanogaster/Gene/Summary?g=FBgn0261360) |  |
