# Supplementary material for: A partial genome assembly of the miniature parasitoid wasp, Megaphragma amalphitanum
Source: PLoS One. 2019 Dec 23;14(12):e0226485. doi: 10.1371/journal.pone.0226485 (PMC6927652; doi:10.1371/journal.pone.0226485)
Supplement: S8 Table — (DOCX) [file pone.0226485.s022.docx]

S8 Table. Common putative venom constituents in Chalcidoidea parasitoid wasps *M. amalphitanum, C. solmsi, M. spermotrophus, T. pretiosum, N. vitripennis.*

| **NP_001155017.1 serine protease 33 precursor** |
| --- |
| **NP_001155147.1 venom acid phosphatase-like precursor** |
| **NP_001155160.1 venom protein F precursor** |
| **NP_001155144.1 gamma-glutamyl cyclotransferase-like venom protein isoform 1 precursor** |
| **NP_001155153.1 aminotransferase-like venom protein 1 precursor** |
| **NP_001155043.1 serine protease 22 precursor** |
| **NP_001155086.1 glucose dehydrogenase-like venom protein** |
| **NP_001155148.1 carboxylesterase clade B, member 2 precursor** |
| **NP_001155076.1 serine protease 50 precursor** |
| **NP_001155015.1 serine protease precursor** |
| **NP_001155040.1 low-density lipoprotein receptor-like venom protein precursor** |
| **NP_001155145.1 gamma-glutamyl cyclotransferase-like venom protein isoform 2** |
| **NP_001154998.1 cysteine-rich/KU venom protein precursor** |
| **NP_001155014.1 serine protease 96 precursor** |
| **NP_001155042.1 serine protease 97 precursor** |
| **NP_001155154.1 antigen 5-like protein 1 precursor** |
| **NP_001155016.1 serine protease homolog 29 precursor** |
| **NP_001155077.1 serine protease 16 precursor** |
| **NP_001155156.1 aminotransferase-like venom protein 2 precursor** |
| **NP_001154991.1 lipase A-like precursor** |
| **NP_001155084.1 chitinase 5 precursor** |
| **NP_001155079.1 serine protease homolog 42 isoform 2 precursor** |
| **NP_001155078.1 serine protease homolog 42 isoform 1 precursor** |
| **NP_001155158.1 venom laccase precursor** |
| **NP_001155164.1 venom protein R precursor** |
| **NP_001155060.1 serine protease homolog 21 precursor** |
| **NP_001155157.1 aspartylglucosaminidase precursor** |
| **NP_001155159.1 laccase-like precursor** |
